# Eicosapentaenoic acid and aspirin, alone and in combination, for the prevention of colorectal adenomas (seAFOod Polyp Prevention trial): a multicentre, randomised, double-blind, placebo-controlled, 2 × 2 factorial trial

**DOI:** 10.1016/S0140-6736(18)31775-6

**Published:** 2018-12-15

**Authors:** Mark A Hull, Kirsty Sprange, Trish Hepburn, Wei Tan, Aisha Shafayat, Colin J Rees, Gayle Clifford, Richard F Logan, Paul M Loadman, Elizabeth A Williams, Diane Whitham, Alan A Montgomery, seAFOod Collaborative Group

**Affiliations:** a Institute of Biomedical and Clinical Sciences, University of Leeds, St James's University Hospital, Leeds, UK; b Nottingham Clinical Trials Unit, School of Medicine, University of Nottingham, Queen's Medical Centre, Nottingham, UK; c Nottingham Digestive Diseases Centre, University of Nottingham, Queen's Medical Centre, Nottingham, UK; d Northern Institute for Cancer Research, Newcastle University, Newcastle-upon-Tyne, UK; e South Tyneside NHS Foundation Trust, South Tyneside District Hospital, Tyne and Wear, UK; f School of Pharmacy and Medical Sciences, Institute of Cancer Therapeutics, University of Bradford, Bradford, UK; g Department of Oncology and Metabolism, University of Sheffield, Sheffield, UK

## Abstract

**Background:**

The omega-3 polyunsaturated fatty acid eicosapentaenoic acid (EPA) and aspirin both have proof of concept for colorectal cancer chemoprevention, aligned with an excellent safety profile. Therefore, we aimed to test the efficacy of EPA and aspirin, alone and in combination and compared with a placebo, in individuals with sporadic colorectal neoplasia detected at colonoscopy.

**Methods:**

In a multicentre, randomised, double-blind, placebo-controlled, 2 × 2 factorial trial, patients aged 55–73 years who were identified during colonoscopy as being at high risk in the English Bowel Cancer Screening Programme (BCSP; ≥3 adenomas if at least one was ≥10 mm in diameter or ≥5 adenomas if these were <10 mm in diameter) were recruited from 53 BCSP endoscopy units in England, UK. Patients were randomly allocated (1:1:1:1) using a secure web-based server to receive 2 g EPA-free fatty acid (FFA) per day (either as the FFA or triglyceride), 300 mg aspirin per day, both treatments in combination, or placebo for 12 months using random permuted blocks of randomly varying size, and stratified by BCSP site. Research staff and participants were masked to group assignment. The primary endpoint was the adenoma detection rate (ADR; the proportion of participants with any adenoma) at 1 year surveillance colonoscopy analysed in all participants with observable follow-up data using a so-called at-the-margins approach, adjusted for BCSP site and repeat endoscopy at baseline. The safety population included all participants who received at least one dose of study drug. The trial is registered with the International Standard Randomised Controlled Trials Number registry, number ISRCTN05926847.

**Findings:**

Between Nov 11, 2011, and June 10, 2016, 709 participants were randomly assigned to four treatment groups (176 to placebo, 179 to EPA, 177 to aspirin, and 177 to EPA plus aspirin). Adenoma outcome data were available for 163 (93%) patients in the placebo group, 153 (85%) in the EPA group, 163 (92%) in the aspirin group, and 161 (91%) in the EPA plus aspirin group. The ADR was 61% (100 of 163) in the placebo group, 63% (97 of 153) in the EPA group, 61% (100 of 163) in the aspirin group, and 61% (98 of 161) in the EPA plus aspirin group, with no evidence of any effect for EPA (risk ratio [RR] 0·98, 95% CI 0·87 to 1·12; risk difference −0·9%, −8·8 to 6·9; p=0·81) or aspirin (RR 0·99 (0·87 to 1·12; risk difference −0·6%, −8·5 to 7·2; p=0·88). EPA and aspirin were well tolerated (78 [44%] of 176 had ≥1 adverse event in the placebo group compared with 82 [46%] in the EPA group, 68 [39%] in the aspirin group, and 76 [45%] in the EPA plus aspirin group), although the number of gastrointestinal adverse events was increased in the EPA alone group at 146 events (compared with 85 in the placebo group, 86 in the aspirin group, and 68 in the aspirin plus placebo group). Six upper-gastrointestinal bleeding events were reported across the treatment groups (two in the EPA group, three in the aspirin group, and one in the placebo group).

**Interpretation:**

Neither EPA nor aspirin treatment were associated with a reduction in the proportion of patients with at least one colorectal adenoma. Further research is needed regarding the effect on colorectal adenoma number according to adenoma type and location. Optimal use of EPA and aspirin might need a precision medicine approach to adenoma recurrence.

**Funding:**

Efficacy and Mechanism Evaluation Programme, a UK Medical Research Council and National Institute for Health Research partnership.

## Introduction

Despite substantial advances in diagnosis and treatment, colorectal cancer remains the second most common cause of cancer death in the UK, causing 16 000 deaths in 2014, with an estimated 1·4 million cases diagnosed worldwide in 2012.^1^

One strategy to reduce colorectal cancer incidence and mortality is prevention. Prevention measures include population screening (by faecal occult blood testing or lower gastrointestinal endoscopy) and endoscopic surveillance of high-risk groups.^2^ However, only 10% of colorectal cancers in the UK are diagnosed within screening programmes.^3^ Moreover, post-colonoscopy colorectal cancers still occur in patients undergoing screening and surveillance, particularly right-sided (proximal) colon cancer.^4^ Therefore, an unmet clinical need for safe and effective colorectal cancer chemoprevention (the use of drugs or nutritional agents) remains, either as a primary measure or in combination with existing surveillance programmes.

**Research in context**

**Evidence before this study**

We searched PubMed for articles published in English between Jan 1, 1990, and Jan 1, 2011, using the terms “colorectal cancer”, “colorectal adenoma”, “chemoprevention”, “omega-3 polyunsaturated fatty acid”, “aspirin”, and “clinical trial”. We found one small randomised trial of eicosapentaenoic acid (EPA) for colorectal cancer chemoprevention in familial adenomatous polyposis and four randomised sporadic polyp prevention trials of aspirin, which used varying doses in different risk populations.

**Added value of this study**

To our knowledge, this is the first randomised trial of EPA for sporadic colorectal cancer chemoprevention and the first evaluation of aspirin in a high-risk population within a high quality-assured national bowel cancer screening programme.

EPA and aspirin did not reduce the proportion of individuals with any colorectal adenoma (adenoma detection rate [ADR]), but they both decreased the recurrence of some subtypes of adenoma, measured by adenoma number, 1 year after clearance screening colonoscopy. There was evidence of selectivity for adenoma type and location.

**Implications of all the available evidence**

Although our trial found no effect on the primary outcome of the proportion of patients with at least one adenoma, both EPA and aspirin show some chemoprevention efficacy for colorectal cancer. The larger effect size of aspirin adds to the weight of evidence for its use in combination with endoscopic screening and surveillance, which provides suboptimal protection against right-sided colorectal cancer.

Our findings suggest that a precision medicine approach (for adenoma type and location) to colorectal cancer chemoprevention will be necessary, which mirrors best practice in colorectal cancer treatment based on molecular stratification.

The trial raises the crucial question of whether ADR or adenoma number is the best measure of chemoprevention efficacy in polyp prevention trials that are based on quality-assured colonoscopy in individuals at high risk of colorectal cancer, in which the ADR is used as a performance indicator, and the adenoma recurrence rate is high.

Colorectal cancer develops over a period of years via a benign lesion termed the colorectal adenoma (also known as a polyp),^5^ which represents a biomarker of subsequent colorectal cancer risk, but is also a clinically important lesion per se, the removal of which by endoscopic polypectomy reduces future colorectal cancer risk.^6^ In parallel with the emergence of a stratified medicine approach to colorectal cancer treatment based on molecular profiling,^7^ the complexity of the early stages of colorectal carcinogenesis is now recognised. This complexity is reflected by two main histological types of precursor lesion (the conventional adenoma and serrated adenoma, which is now termed sessile serrated lesion, recognising that no dysplasia is present in most serrated polyps),^8^ which map differently against molecular characteristics including chromosomal instability, microsatellite instability, and CpG island DNA hypermethylation (CIMP).^9^

The naturally occurring omega-3 polyunsaturated fatty acid (PUFA) C20:5*n*3 eicosapentaenoic acid (EPA) is licensed for treatment of severe hypertriglyceridaemia that is unresponsive to other therapies and has clinical proof-of-concept as a colorectal cancer chemoprevention agent from a randomised controlled trial (RCT) in patients with familial adenomatous polyposis, in which 2 g EPA free fatty acid (FFA) per day for 6 months was associated with a significant reduction in rectal adenoma number and size.^10^ Omega-3 PUFAs have been shown to have an excellent safety and tolerability profile on the basis of vast cardiology experience.^11^

A robust body of evidence exists from polyp prevention RCTs in individuals undergoing colonoscopic surveillance after colorectal adenoma clearance,^12^ as well as long-term follow-up of colorectal cancer outcomes after aspirin RCTs with vascular endpoints, that aspirin prevents colorectal cancer.^13^ However, aspirin has not been adopted for sporadic colorectal cancer chemoprevention to date, because of uncertainty about dose and the most appropriate target population balancing colorectal cancer risk and adverse event (ie, bleeding) profile.^14^

The National Health Service (NHS) Bowel Cancer Screening Programme (BCSP) in England provides population-level colorectal cancer screening using guaiac faecal occult blood testing, high quality-assured screening colonoscopy, and surveillance on the basis of colorectal adenoma number and characteristics,^2^ thus providing an excellent opportunity for an RCT of chemoprevention in individuals at high risk of future colorectal neoplasia.

Colorectal adenoma risk reduction has previously been shown at 1 year in polyp prevention RCTs of aspirin and metformin.^12^, ^15^, ^16^ Therefore, we aimed to test the chemoprevention efficacy of EPA and aspirin, alone and in combination, in individuals with high-risk colorectal adenoma features at screening colonoscopy, who subsequently had a surveillance colonoscopy at 1 year, in the BCSP.

## Methods

### Study design and participants

The Systematic Evaluation of Aspirin and Fish Oil (seAFOod) Polyp Prevention Trial was a multicentre, randomised, double-blind, placebo-controlled, 2 × 2 factorial trial. The trial protocol has been published elsewhere.^17^ Approval was obtained from the Trent Research Ethics Committee (10/H0405/90). The seAFOod trial was done in 53 hospital endoscopy units in the English BCSP using its strict protocol-driven and quality-assured practice and reporting mechanisms.^2^ The trial gained approval from the English BCSP Research Committee.

Individuals aged 55–73 years who were identified as high risk at a complete BCSP screening colonoscopy (three or more colorectal adenomas with at least one ≥10 mm in diameter, or five or more colorectal adenomas that were <10 mm in diameter) were screened for trial eligibility. For participants who had a bowel scope flexible sigmoidoscopy in the BCSP, eligibility was established on the basis of combined colorectal adenoma findings from the flexible sigmoidoscopy and subsequent colonoscopy.

All individuals at high risk were provided with written trial information after colonoscopy and then attended a routine BCSP outpatient visit 7–14 days later, during which written informed consent was obtained if they were eligible. Exclusion criteria included requirement for more than one repeat colonoscopy or flexible sigmoidoscopy within the 3 month BCSP screening window, regular (>3 doses per week) prescribed or over-the-counter (OTC) aspirin or non-aspirin non-steroidal anti-inflammatory drug (NSAID) use, and concomitant warfarin therapy or use of any other anticoagulant or antiplatelet agent.^17^ A complete list of all inclusion and exclusion criteria can be found in the protocol summary on the International Standard Randomised Controlled Trials Number (ISRCTN) registry.

### Randomisation and masking

Participants were randomly allocated in a 1:1:1:1 ratio to either EPA plus aspirin; EPA plus placebo; aspirin plus placebo; or placebo plus placebo. Randomisation occurred within 4 weeks from the screening colonoscopy by research staff at each site using a secure web-based system with treatment assignment established by a pseudorandom code, using random permuted blocks of randomly varying size. Stratification was done by BCSP site.

The sequence of treatment allocations was concealed until recruitment, data collection, and database lock were completed. Investigational medicinal product (IMP) allocation was not divulged to any research staff or participant.

### Procedures

Participants who were randomly assigned to receive EPA received either 2 g 99% EPA-FFA per day (as two 500 mg gastro-resistant capsules taken twice per day with food, supplied by SLA Pharma AG, Liestal, Switzerland), as used in the previous RCT in patients with familial adenomatous polyposis,^10^ or an equivalent FFA dose as 2780 mg 90% EPA-triglyceride per day (as five soft gelatine capsules per day split between two meals, purchased from Igennus Healthcare Nutrition, Cambridge, UK; appendix). Those assigned to receive a placebo of EPA received the respective identical-looking capsules (both containing mixed capric and capryllic acid medium-chain triglycerides). Participants also received either one 300 mg enteric-coated aspirin tablet per day with food or an identical placebo tablet (both supplied by Bayer AG, Leverkusen, Germany). Capsule and tablet were taken until the day before surveillance (exit) colonoscopy, which was scheduled 12 months after screening. During the intervention phase, SLA Pharma were unable to supply sufficient EPA-FFA capsules, which resulted in a switch to the EPA-triglyceride formulation (appendix). Each participant received only one formulation of EPA (either FFA or triglyceride) or its matching placebo.

Participants could withdraw from the trial at any time. When a participant withdrew from the intervention only, follow-up occurred per protocol. If a participant withdrew from the trial, data collected up to the point of withdrawal were included in the analyses.

Participants completed a European Prospective Investigation into Cancer and Nutrition short food frequency questionnaire at study entry and after surveillance colonoscopy at 12 months, for categorisation of total and oily fish intake (appendix). A prescription was issued for the supply of the IMP by the local hospital pharmacy at randomisation and at clinical review at 6 months. Blood (for plasma, red blood cells, and leucocytes) and urine samples were obtained at study entry, at 6 months, and at surveillance colonoscopy. Rectal mucosa (four biopsy samples of macroscopically normal mucosa ≤2 cm from any polyp) was collected at the end of the surveillance colonoscopy.^17^ Telephone consultations were done at 2 weeks, 12 weeks, and 38 weeks after randomisation to monitor for adverse events (with emphasis on emergent gastrointestinal symptoms), check concomitant medication use, and confirm IMP compliance. Participants scheduled for repeat lower gastrointestinal endoscopy stopped the IMP 10 days before and restarted IMP 4 days after to minimise potential bleeding risk.

Fatty acids were extracted from red blood cell membranes and rectal mucosa and then measured by liquid chromatography tandem mass spectrometry as previously described.^18^ Data are expressed as the percentage of each fatty acid relative to the total fatty acid chromatographic peak area.^18^

Colorectal adenoma outcomes at the 12 month surveillance colonoscopy were collected as per routine BCSP practice,^2^, ^19^ including the number, size (maximum dimension in millimetres as indicated on the histopathology report or the endoscopic size if the adenoma was not retrieved or was removed by hot biopsy), site (proximal to the splenic flexure [right] or distal to the splenic flexure [left]), histological type (tubular or tubulovillous, villous, or serrated—not including hyperplastic polyps]), and presence of high-grade dysplasia for all colorectal adenomas.

### Outcomes

The primary outcome was the proportion of participants with one or more colorectal adenomas detected at surveillance colonoscopy—ie, the adenoma detection rate (ADR; centrally assessed). The secondary outcomes were as follows: the proportion of participants with advanced (≥10 mm diameter, high-grade dysplasia, or villous histology) colorectal adenomas and with conventional (this term encompassing tubular, tubulovillous, and villous adenomas was adopted only after database lock), serrated, left-sided, and right-sided colorectal adenoma subtypes (serrated adenoma was added as a secondary endpoint to the SAP before database lock); the mean number of colorectal adenomas per participant for all colorectal adenomas, and for advanced, conventional, serrated, left-sided, and right-sided colorectal adenoma subtypes; the number of participants at high risk who were reclassified as being at intermediate risk (BCSP guidelines mandate that any individual who does not continue to fulfil high-risk criteria should be classified as being at intermediate risk for 3-year surveillance colonoscopy); development of colorectal cancer; dietary fish and other seafood intake at baseline and at 12 months; red blood cell and rectal mucosal PUFA concentrations; and adverse events, including clinically significant bleeding episodes (haemorrhagic stroke or gastrointestinal bleeding).^17^ Post-hoc exploratory analyses were done on colorectal adenoma size, the relationship between individual rectal mucosal EPA levels and colorectal adenoma number, and the association between rectal mucosal and red blood cell EPA levels at 12 months. The analysis of colorectal adenoma size was based on the within-participant mean colorectal adenoma size and was adjusted for histological subtype (conventional or serrated) and BCSP site. A full list of all endpoints can be found in the protocol summary on the ISRCTN registry.

### Statistical analysis

A sample size of 768 evaluable individuals defined as high risk by BCSP allowed detection of a minimum 18% relative (10% absolute) reduction in ADR from 60% for both agents within a 2 × 2 factorial design, assuming independent intervention effects, with 80% power and a 5% two-sided significance level, as described in the protocol.^17^ This effect size was less than that observed in the previous RCT of EPA-FFA in patients with familial adenomatous polyposis,^10^ and lower than the relative ADR reduction in the meta-analysis of aspirin polyp prevention trials.^12^ Allowing for 10% of participants to drop out, the target number of participants to be randomly assigned was 853.

Primary and secondary outcomes were analysed according to allocation, regardless of compliance with treatment, for all participants who were randomly assigned and who had observed follow-up data. Per-protocol analysis of the primary outcome was based on all participants who were randomly assigned without a major protocol violation (ie, intake of ≤75% capsules or ≤50% tablets, any use of OTC medication containing aspirin, NSAIDs, or fish oil for >2 weeks, or ineligibility after randomisation) before treatment codes were revealed. Adverse events are reported in the safety population of all participants who were randomly assigned and who received at least one dose of IMP.

The primary outcome was analysed with a so-called at-the-margins approach,^20^ after first examining whether there was any evidence of an interaction between EPA and aspirin. The log relative risk was estimated using a mixed effects log-binomial regression model, with BCSP site as a random effect and the risk difference and ratio presented with the 95% CI. Both interventions were fitted simultaneously and the analysis was adjusted according to whether a repeat endoscopic screening procedure was required and according to BCSP site. Sensitivity analyses were done to support the primary analysis. All secondary analyses assumed that data were missing at random and no imputation was done. Data are presented as the risk difference for the binary ADR outcomes and the incidence rate ratio (IRR) for continuous data (ie, the mean number of adenomas per participant).

Treatment-emergent adverse events and adverse drug reactions were summarised by MedDRA system organ class. Gastrointestinal adverse events were also summarised by MedDRA preferred term and EPA formulation (FFA or triglyceride). The worst case (severe event or event related to trial treatment) was assumed if severity or causality data were missing. Clinically significant bleeding episodes (haemorrhagic stroke or acute upper gastrointestinal bleeding requiring hospital admission or investigation) were identified by the chief investigator (MAH) using a manual search of all adverse events. Common gastrointestinal adverse events were presented separately as clinically meaningful symptom categories, as defined by the chief investigator.

The statistical analysis plan was finalised before database lock on Aug 31, 2017. The plan included comparison of the EPA formulations (safety and tolerability, as well as EPA incorporation), analysis of serrated adenoma outcomes, and definition of the analysis population to include only participants with observed follow-up data, compared with the analysis described in the protocol.^17^ Extra sensitivity analyses of the primary outcome (BCSP centre and site as random effects in a multilevel model, without adjustment for repeat endoscopy, baseline red blood cell EPA levels, and oily fish intake), conventional colorectal adenoma outcomes, and the aforementioned exploratory analyses were later added.

All statistical analyses were done using Stata version 15.0. The trial is registered with the ISRCTN registry, number ISRCTN05926847.

### Role of the funding source

The funder of the study had no role in study design, data collection, data analysis, data interpretation, or writing of the report. The corresponding author had full access to all the data in the study and had final responsibility for the decision to submit for publication.

## Results

**Figure 1:**
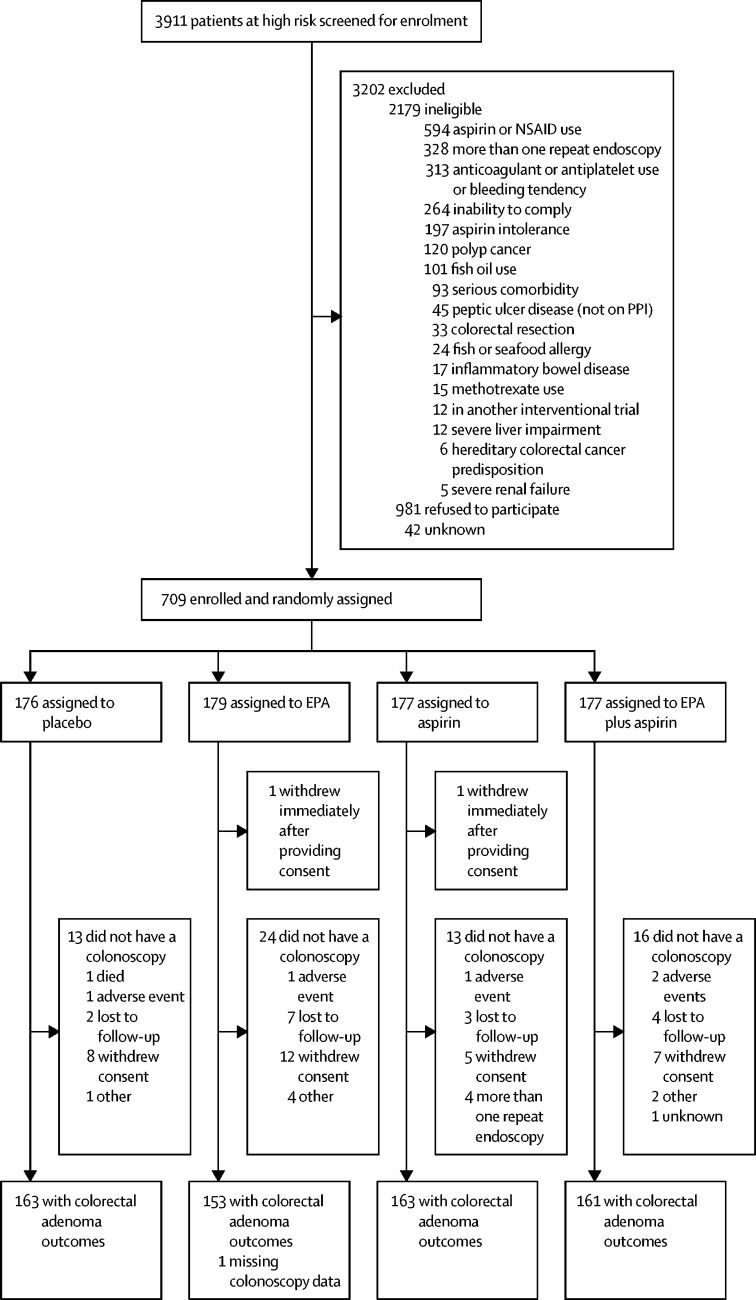
Trial profile EPA=eicosapentaenoic acid. NSAID=non-steroidal anti-inflammatory drug. PPI=proton-pump inhibitor.

Patients were recruited between Nov 11, 2011, and June 10, 2016 (mandated by the maximum shelf life of EPA-triglyceride specified by the Medicines and Healthcare Products Regulatory Agency; appendix). During this period, 3911 individuals who were defined as high risk by the BCSP were identified and assessed for eligibility, of whom 3202 (82%) were excluded (figure 1). Existing aspirin or other NSAID use occurred in 594 (15%) screened individuals at high risk, and concurrent anticoagulant or non-aspirin antiplatelet agent use was an exclusion criterion in 313 (8%) patients. Overall, 709 (18%) patients were randomly assigned, 176 to placebo, 179 to EPA alone, 177 to aspirin alone, and 177 to EPA plus aspirin. 641 (90%) patients had a surveillance colonoscopy, with colorectal adenoma outcome data available for 640 (90%) individuals (figure 1). 422 (60%) participants were randomly assigned to active EPA-FFA or placebo (109 to placebo, 108 to EPA, 101 to aspirin, and 104 to EPA plus aspirin) and 287 (40%) participants were randomly assigned to active EPA-triglyceride or placebo (67 to placebo, 71 to EPA, 76 to aspirin, and 73 to EPA plus aspirin).

Baseline characteristics were well balanced across the four treatment groups (table 1). The mean age of participants was 65 years (IQR 62–69) and 80% were men, reflecting the high-risk patient demographic in the English BCSP.^17^ We noted a slight imbalance across groups with respect to smoking status, with fewer current smokers present in the EPA treatment group. A history of abdominal pain or dyspepsia was reported by 158 (22%) of 707 participants (36 [20%] in the placebo group, 40 [22%] in the EPA group, 41 [23%] in the aspirin group, and 41 [23%] in the EPA plus aspirin group).

**Table 1:** Baseline characteristics

			**Placebo (n=176)**	**EPA (n=178)**	**Aspirin (n=176)**	**EPA plus aspirin (n=177)**	**Total (n=707)**
Age (years)			65 (62–69)	65 (62–69)	65 (62–69)	66 (62–69)	65 (62–69)
Sex							
	Male		139 (79%)	138 (78%)	140 (80%)	146 (82%)	563 (80%)
	Female		37 (21%)	40 (22%)	36 (20%)	31 (18%)	144 (20%)
Excess bodyweight*							
	Overweight (BMI 25–29·9)		76 (43%)	77 (43%)	81 (46%)	77 (44%)	311 (44%)
	Obese (BMI ≥30)		68 (39%)	70 (39%)	71 (40%)	61 (34%)	270 (38%)
	History of diabetes		24 (14%)	24 (13%)	18 (10%)	15 (8%)	81 (11%)
Cigarette smoking							
	Current smoker		34 (19%)	13 (7%)	27 (15%)	32 (18%)	106 (15%)
	Previous smoker		82 (47%)	96 (54%)	89 (51%)	80 (45%)	347 (49%)
	Never smoked		60 (34%)	69 (39%)	60 (34%)	65 (37%)	254 (36%)
Regular medication			81 (46%)	93 (52%)	88 (50%)	92 (52%)	354 (50%)
Medication before trial entry†							
	Statin		50 (28%)	54 (30%)	51 (29%)	55 (31%)	210 (30%)
	Calcium		1 (1%)	3 (2%)	3 (2%)	0	7 (1%)
	Calcium plus vitamin D		2 (1%)	1 (<1%)	4 (2%)	4 (2%)	11 (2%)
	Metformin		14 (8%)	12 (7%)	11 (6%)	9 (5%)	46 (7%)
	Glitazone		1 (<1%)	1 (<1%)	1 (<1%)	0	3 (<1%)
	Proton-pump inhibitor		19 (11%)	27 (15%)	24 (14%)	20 (11%)	90 (13%)
	Aspirin		0	0	0	1 (<1%)	1 (<1%)
	Fish oil		1 (1%)	4 (2%)	2 (1%)	2 (1%)	9 (1%)
	Non-aspirin NSAID		1 (1%)	4 (2%)	1 (<1%)	5 (3%)	11 (2%)
	Other		34 (19%)	34 (19%)	37 (21%)	48 (27%)	153 (22%)
Colorectal adenoma characteristics							
	Total number of adenomas		856	892	927	856	3531
	Number of adenomas per participant		4·9 (2·6)	5·0 (2·2)	5·3 (2·7)	4·8 (2·3)	5·0 (2·5)
	Number of advanced‡ adenomas per participant		1·2 (0·9)	1·3 (1·0)	1·2 (0·9)	1·1 (0·9)	1·2 (0·9)
	≥1 adenoma proximal to splenic flexure		141 (80%)	146 (82%)	153 (87%)	144 (81%)	584 (83%)
	Histological type§						
		Conventional	812/856 (95%)	844/892 (95%)	895/927 (97%)	809/856 (95%)	3360 (95%)
		Tubular or tubulovillous	807/856 (94%)	834/892 (93%)	885/927 (95%)	803/856 (94%)	3329 (94%)
		Villous	5/856 (1%)	10/892 (1%)	10/927 (1%)	6/856 (<1%)	31 (1%)
		Serrated	22/856 (3%)	30/892 (3%)	18/927 (2%)	21/856 (2%)	91 (3%)
		Not sent to pathology	18/856 (2%)	16/892 (2%)	13/927 (2%)	21/856 (2%)	68 (2%)
		Missing data	4/856 (<1%)	2/892 (<1%)	1/927 (<1%)	5/856 (1%)	12 (<1%)
Repeat screening endoscopy							
	No		136 (77%)	128 (72%)	133 (76%)	133 (75%)	530 (75%)
	Yes		25 (14%)	33 (19%)	24 (14%)	34 (19%)	116 (16%)
	Missing		15 (9%)	17 (10%)	19 (11%)	10 (6%)	61 (9%)

One participant in the EPA group and one in the aspirin group withdrew from the study immediately after providing consent and so their data are not reported here. Data are median (IQR), n (%), or mean (SD). BMI=body-mass index. EPA=eicosapentaenoic acid. NSAID=non-steroidal anti-inflammatory drug.

* As per WHO guidelines, BMI is measured as kg/m^2^.

† Not mutually exclusive, some participants reported more than one category.

‡ Diameter of at least 10 mm, high-grade dysplasia, or tubulovillous or villous histology.

§ Colorectal adenoma-level data.

Screening colonoscopy findings were balanced across the treatment groups (table 1). Compliance with both capsule and tablet IMPs was excellent, with mean compliance percentage as measured by capsule and tablet counting being 94–97% (appendix). During the intervention phase, 18 (26%) of 707 participants started other regular medication, which was balanced across treatment groups (appendix). Dietary fish intake was similar across treatment groups (appendix), with no discernible difference between the groups regarding the proportion of people who changed their dietary fish intake during the intervention (appendix).

**Figure 2:**
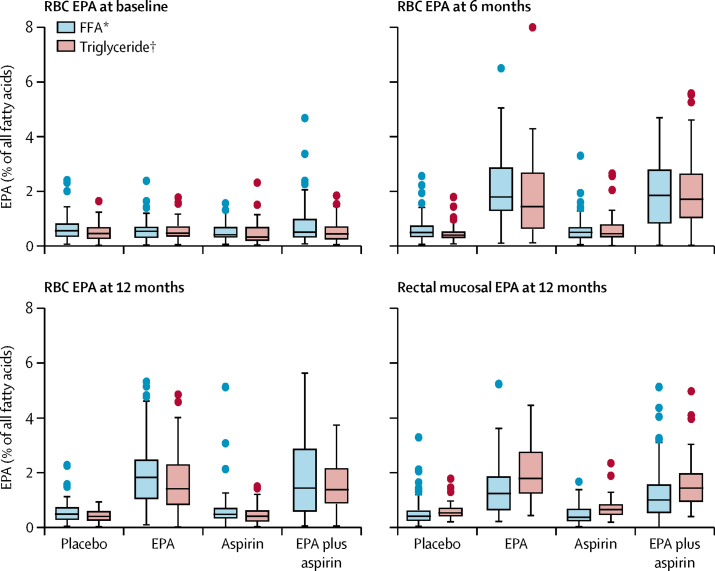
RBC and rectal mucosal EPA concentrations by trial group and EPA formulation Boxes represent the median and IQR. Whiskers represent 1·5 times the IQR with outlier values (individual datapoints) above and below the IQR. EPA=eicosapentaenoic acid. FFA=free-fatty acid. RBC=red blood cell. *Individuals randomly assigned to EPA–FFA or its placebo. †Individuals randomised to EPA–triglyceride or its placebo.

As expected, individuals in the EPA treatment group showed an increase in red blood cell and rectal mucosal EPA content compared with baseline values, unlike the trial groups that did not include active EPA treatment (figure 2). Comparison of red blood cell and rectal mucosal EPA concentrations during the intervention and comparison of the change from baseline values in individuals who received active EPA capsules confirmed that EPA incorporation during the trial was similar between participants who were allocated FFA or triglyceride capsules (figure 2; appendix). No evidence of conversion from EPA to the other main bioactive long-chain omega-3 PUFA C22:6*n*3 docosahexaenoic acid was observed, and there was no evidence of a clear reduction in tissue C20:4*n*6 arachidonic acid content, in red blood cells or in the rectum (appendix). The ratio of EPA to arachidonic acid, which is often reported as a therapeutic response biomarker of EPA in cardiovascular trials,^11^ was similar in red blood cells and the rectum of participants who received either FFA or triglyceride (appendix).

The median time between randomisation and surveillance colonoscopy was 344–349 days (table 2). The primary outcome (total ADR) was similar across the four treatment groups (100 [61%] of 163 patients in the placebo group *vs* 97 [63%] of 153 in the EPA group *vs* 100 [61%] of 163 in the aspirin group *vs* 98 [61%] of 161 in the EPA plus aspirin group; table 2). There was no evidence of an interaction between EPA and aspirin for the ADR (interaction risk difference −1·5%, 95% CI −17·1 to 14·2, p_interaction_=0·8530). Therefore, the ADR was analysed according to factorial margins (table 3). The risk difference and risk ratio of having at least one colorectal adenoma did not differ between EPA and placebo, and it also did not differ between aspirin and placebo (table 4; appendix). The 95% CI for the risk difference for both interventions did not exceed −10% (the absolute ADR difference that the trial was powered to detect). An analysis that was not adjusted for repeat colonoscopy showed similar results (appendix). Multiple sensitivity analyses, including per-protocol analysis, were supportive of the primary analysis (appendix).

**Table 2:** Primary and secondary colorectal adenoma outcomes, per treatment group

		**Placebo (n=163)**	**EPA (n=153)**	**Aspirin (n=163)**	**EPA plus aspirin (n=161)**
**Total adenoma detection rate**					
Time from randomisation to colonoscopy (days)		344 (334–360)	349 (333–363)	348 (335–364)	348 (337–364)
Participants with ≥1 colorectal adenoma		100 (61%)	97 (63%)	100 (61%)	98 (61%)
**Total colorectal adenomas**					
Overall number of colorectal adenomas		231	238	209	166
Overall number of advanced colorectal adenomas		12	8	11	9
Histology of colorectal adenomas					
	Conventional	220 (95%)	205 (86%)	194 (93%)	155 (93%)
	Serrated	8 (3%)	21 (9%)	10 (5%)	4 (2%)
	Missing data	3 (1%)	12 (5%)	5 (2%)	7 (4%)
Location of colorectal adenomas					
	Left	93 (40%)	98 (41%)	101 (48%)	58 (35%)
	Right	138 (60%)	140 (59%)	107 (51%)	108 (65%)
	Missing data	0	0	1 (<1%)	0
Total number of colorectal adenomas per participant					
	Mean (SD)	1·4 (2·0)	1·6 (2·1)	1·3 (1·6)	1·0 (1·2)
	Minimum	0	0	0	0
	Maximum	16	10	13	6
Incidence of colorectal adenomas per person per year					
	Mean (SD)	1·5 (2·1)	1·6 (2·2)	1·3 (1·7)	1·1 (1·3)
	Minimum	0	0	0	0
	Maximum	16·6	10·8	13·5	6·7
**Advanced colorectal adenomas**					
Participants with ≥1 advanced colorectal adenoma		11 (7%)	8 (5%)	10 (6%)	8 (5%)
Number of advanced colorectal adenomas per participant					
	Mean (SD)	0·1 (0·3)	0·1 (0·2)	0·1 (0·3)	0·1 (0·3)
	Minimum	0	0	0	0
	Maximum	2	1	2	2
Incidence of advanced colorectal adenomas per person per year					
	Mean (SD)	0·1 (0·3)	0·1 (0·2)	0·1 (0·3)	0·1 (0·3)
	Minimum	0	0	0	0
	Maximum	2·1	1·2	2·1	2·1
**Conventional colorectal adenomas**					
Participants with ≥1 conventional colorectal adenoma		92 (56%)	83 (54%)	91 (56%)	88 (55%)
Number of conventional colorectal adenomas per participant					
	Mean (SD)	1·4 (2·0)	1·4 (1·9)	1·2 (1·6)	1·0 (1·2)
	Minimum	0	0	0	0
	Maximum	16	10	13	6
Incidence of conventional colorectal adenomas per person per year					
	Mean (SD)	1·4 (2·1)	1·4 (2·0)	1·2 (1·7)	1·0 (1·3)
	Minimum	0	0	0	0
	Maximum	16·6	10·8	13·5	6·7
**Serrated colorectal adenomas**					
Participants with ≥1 serrated colorectal adenoma		7 (4%)	11 (7%)	6 (4%)	4 (2%)
Number of serrated colorectal adenomas per participant					
	Mean (SD)	0 (0·2)	0·1 (0·7)	0·1 (0·4)	0 (0·2)
	Minimum	0	0	0	0
	Maximum	2	8	4	1
Incidence of serrated colorectal adenomas per person per year					
	Mean (SD)	0·1 (0·3)	0·1 (0·8)	0·1 (0·3)	0 (0·2)
	Minimum	0	0	0	0
	Maximum	2·2	8·6	3·4	1·1
**Left-sided colorectal adenomas**					
Participants with ≥1 left-sided colorectal adenoma		55 (34%)	58 (38%)	65 (40%)	42 (26%)
Number of left-sided colorectal adenomas per participant					
	Mean (SD)	0·6 (1·0)	0·6 (1·1)	0·6 (0·9)	0·4 (0·7)
	Minimum	0	0	0	0
	Maximum	5	5	5	3
Incidence of left-sided colorectal adenomas per person per year					
	Mean (SD)	0·6 (1·0)	0·7 (1·1)	0·6 (0·9)	0·4 (0·7)
	Minimum	0	0	0	0
	Maximum	5·6	5·4	4·4	3·3
**Right-sided colon adenomas**					
Participants with ≥1 right-sided colon adenoma		66 (40%)	72 (47%)	63 (39%)	69 (43%)
Number of right colon adenomas per participant					
	Mean (SD)	0·8 (1·7)	0·9 (1·5)	0·7 (1·3)	0·7 (1·0)
	Minimum	0	0	0	0
	Maximum	16	9	13	6
Incidence of right-sided colon adenomas per person per year					
	Mean (SD)	0·9 (1·8)	1·0 (1·6)	0·7 (1·4)	0·7 (1·1)
	Minimum	0	0	0	0
	Maximum	16·6	10·4	13·5	6·7
**Future risk stratification**					
Participants reclassified as intermediate risk		147 (90%)	128 (84%)	140 (86%)	146 (91%)

Data are median (IQR), number, number (%), or mean (SD), unless otherwise specified. EPA=eicosapentaenoic acid.

**Table 3:** Primary and secondary colorectal adenoma outcomes, by factorial margins

		**EPA (n=314)**	**No EPA (n=326)**	**Aspirin (n=324)**	**No aspirin (n=316)**
Participants with ≥1 colorectal adenoma		195 (62%)	200 (61%)	198 (61%)	197 (62%)
Incidence of colorectal adenomas per person per year					
	Mean (SD)	1·3 (1·8)	1·4 (1·9)	1·2 (1·5)	1·6 (2·1)
	Minimum	0	0	0	0
	Maximum	10·8	16·6	13·5	16·6
Incidence of advanced colorectal adenomas per person per year					
	Mean (SD)	0·1 (0·3)	0·1 (0·3)	0·1 (0·3)	0·1 (0·3)
	Minimum	0	0	0	0
	Maximum	2·1	2·1	2·1	2·1
Incidence of conventional colorectal adenomas per person per year					
	Mean (SD)	1·2 (1·7)	1·3 (1·9)	1·1 (1·5)	1·4 (2·0)
	Minimum	0	0	0	0
	Maximum	10·8	16·6	13·5	16·6
Incidence of serrated colorectal adenomas per person per year					
	Mean (SD)	0·1 (0·6)	0·1 (0·3)	0 (0·3)	0·1 (0·6)
	Minimum	0	0	0	0
	Maximum	8·6	3·4	3·4	8·6
Incidence of left-sided colorectal adenomas per person per year					
	Mean (SD)	0·5 (0·9)	0·6 (1·0)	0·5 (0·8)	0·6 (1·1)
	Minimum	0	0	0	0
	Maximum	5·4	5·6	4·4	5·6
Incidence of right-sided colon adenomas per person per year					
	Mean (SD)	0·8 (1·4)	0·8 (1·6)	0·7 (1·3)	0·9 (1·7)
	Minimum	0	0	0	0
	Maximum	10·4	16·6	13·5	16·6

Data are number (%) or mean (SD), unless otherwise specified. EPA=eicosapentaenoic acid.

**Table 4:** Comparison of the adenoma detection rate and adenoma number for the primary and secondary colorectal adenoma outcomes

	**EPA *vs* no EPA**	**Aspirin *vs* no aspirin**
**All colorectal adenomas**		
Risk difference (95% CI)*	−0·9% (−8·8 to 6·9); p=0·81	−0·6% (−8·5 to 7·2); p=0·88
Risk ratio (95% CI)*	0·98 (0·87 to 1·12)	0·99 (0·87 to 1·12)
IRR (95% CI)	0·91 (0·79 to 1·05)	0·78 (0·68 to 0·90)
**Advanced colorectal adenomas**		
Risk difference (95% CI)	−0·6% (−4·4 to 3·1)	−0·3% (−4·1 to 3·5)
IRR (95% CI)	0·82 (0·43 to 1·56)	0·99 (0·52 to 1·86)
**Conventional colorectal adenomas**		
Risk difference (95% CI)	−3·3% (−11·2 to 4·7)	1·7% (−6·2 to 9·6)
IRR (95% CI)	0·86 (0·74 to 0·99)	0·82 (0·71 to 0·94)
**Serrated colorectal adenomas**		
Risk difference (95% CI)	0% (−3·2 to 3·2)	−2·7% (−6·1 to 0·7)
IRR (95% CI)	1·44 (0·79 to 2·60)	0·46 (0·25 to 0·87)
**Left-sided colorectal adenomas**		
Risk difference (95% CI)	−7·8% (−15·5 to −0·2)	−1·8% (−9·4 to 5·8)
IRR (95% CI)	0·75 (0·60 to 0·94)	0·85 (0·69 to 1·06)
**Right-sided colon adenomas**		
Risk difference (95% CI)	6·0% (−1·9 to 13·9)	−3·1% (−11·0 to 4·7)
IRR (95% CI)	1·02 (0·85 to 1·22)	0·73 (0·61 to 0·88)
**Participants reclassified as intermediate risk**		
Risk difference (95% CI)	−0·2% (−5·4 to 5·1)	0·9% (−14·1 to 6·2)

EPA=eicosapentaenoic acid.

* Adjusted by site as random effect and repeat colonoscopy at baseline, n=588 (152 in the placebo group, 138 in the EPA group, 147 in the aspirin group, and 151 in the EPA plus aspirin group).

Summary data for the number of colorectal adenomas detected at surveillance colonoscopy are shown in table 2 and in the appendix. Numerically fewer colorectal adenomas were detected in individuals who were randomly assigned to combined EPA and aspirin treatment than to the other three groups (table 2), which was most marked for left-sided lesions. The total mean number of colorectal adenomas per participant was 1·4 (SD 2·0) in individuals who were allocated placebo only, compared with 1·6 (2·1) for those allocated EPA and 1·3 (1·6) for those assigned to aspirin. Participants assigned to both EPA and aspirin had a mean of 1·0 (1·2) adenomas per person (table 2). Analysis at the margins revealed an IRR of 0·91 (95% CI 0·79–1·05) for EPA and 0·78 (95% CI 0·68–0·90) for aspirin (table 4).

Secondary ADR outcomes are described in Table 2, Table 3, Table 4 and in the appendix. Few advanced colorectal adenomas were detected (table 2). IRRs from an at-the-margins analysis of the mean number of adenomas per participant for colorectal adenoma subtypes showed an effect of EPA on left-sided and conventional colorectal adenomas, but no evidence of an effect on right-sided or serrated lesions was observed (Table 3, Table 4, appendix). By contrast, aspirin treatment was associated with a statistically significant decrease in the incidence of conventional colorectal adenomas, but also a decrease in IRR for right-sided and serrated lesions (table 3, appendix). In keeping with the primary ADR outcome, no evidence of any difference in colorectal adenoma subtype ADRs for either EPA or aspirin was observed, except for a possible effect of EPA on recurrence of left-sided colorectal adenomas (table 4; appendix). The number of individuals who were reclassified as being at intermediate risk requiring a subsequent 3 year procedure, rather than continuing annual surveillance, was no different across the treatment groups (Table 2, Table 4; appendix). No colorectal cancers were detected during the seAFOod trial.

The safety population comprised 697 participants who received at least one dose of IMP (table 5). Overall, there were no safety concerns in participants receiving either EPA or aspirin (appendix). The majority of adverse events were mild in severity in each of the treatment groups (table 5). An excess of adverse events was observed in participants receiving EPA alone, adverse events that were predominantly gastrointestinal (diarrhoea, abdominal pain, and nausea; appendix) and were explained mainly by clustering of numerous gastrointestinal adverse events in a small number of individuals (table 5). By contrast, the frequency and distribution of gastrointestinal adverse events was similar across the other three groups, including those who received both EPA and aspirin (table 5; appendix). No consistent differences in gastrointestinal adverse events between individuals receiving EPA–FFA or EPA–triglyceride were observed (appendix). The same pattern was noted for adverse drug reactions (appendix). A small number of serious adverse events (SAEs) were reported (table 5; appendix). The most frequently reported SAEs were cardiac events (including three episodes of atrial fibrillation in one participant and one atrial fibrillation episode in two participants, all of whom were receiving EPA alone) and gastrointestinal disorders. No haemorrhagic strokes were reported. Six acute upper-gastrointestinal bleeding events were reported, and were spread across the treatment groups (two in the EPA group, three in the aspirin group, and one in the placebo group). One unrelated death in the placebo group was reported after 9 months of intervention (bladder cancer).

**Table 5:** Safety and tolerability

		**Placebo (n=176)**	**EPA (n=177)**	**Aspirin (n=174)**	**EPA plus aspirin (n=170)**
Participants with one or more adverse events		78 (44%)	82 (46%)	68 (39%)	76 (45%)
Total number of adverse events		160	211	154	129
Severity					
	Mild	119 (74%)	161 (76%)	122 (79%)	110 (85%)
	Moderate	33 (21%)	47 (22%)	28 (18%)	18 (14%)
	Severe	5 (3%)	2 (1%)	4 (3%)	1 (1%)
	Missing	3 (2%)	1 (<1%)	0	0
Participants with one or more SAEs		13 (7%)	12 (7%)	12 (7%)	5 (3%)
Total number of SAEs		16	16	17	6
Participants with one or more gastrointestinal adverse events		51 (29%)	67 (38%)	44 (25%)	47 (28%)
Total number of gastrointestinal adverse events		85	146	86	68
Number of gastrointestinal adverse events per participant					
	One	32	29	20	30
	Two	10	17	13	14
	Three	6	9	7	2
	Four	1	8	2	1
	Five	1	3	1	0
	More than five	1	1	1	0

All data are n (%) unless otherwise indicated. EPA=eicosapentaenoic acid. SAEs=serious adverse events.

No evidence of a difference in colorectal adenoma size associated with either EPA or aspirin use was found (appendix).

Finally, the detailed PUFA measurements done to compare tissue EPA incorporation during dosing with EPA–FFA and EPA–triglyceride provided an opportunity to explore the relationship between target organ (rectal) EPA incorporation and red blood cell EPA levels commonly used as a biomarker of tissue EPA exposure.^18^ A moderate strength correlation (*r*=0·455) between red blood cell and rectal mucosal EPA concentrations was observed at 12 months (appendix). We also did an unplanned investigation of the relationship between individual rectal mucosal EPA concentration and number of colorectal adenomas (total and subtypes) descriptively, comparing EPA users and those who received placebo EPA (appendix). Both EPA users and those who did not use EPA with a higher rectal mucosal EPA concentration appeared to have, in general, a lower colorectal adenoma count than those with a lower rectal mucosal EPA level, which was most marked for total colorectal adenoma number and conventional adenomas (appendix).

## Discussion

The seAFOod Polyp Prevention trial found no evidence of an effect of either EPA or aspirin on the primary outcome of the proportion of individuals with one or more colorectal adenomas at 12 month surveillance colonoscopy (the ADR) in patients defined as high risk by the English BCSP. However, secondary analyses of the effects of EPA and aspirin on colorectal adenoma number provided evidence of the chemopreventive activity of both agents. Aspirin was effective at reducing the total number of colorectal adenomas per participant, but the effect of EPA on total number of adenomas per participant was insufficiently precise to draw a firm conclusion about efficacy. Other secondary analyses suggested that EPA and aspirin have colorectal adenoma subtype-selective and site-selective effects. Participants randomly assigned to receive EPA had a reduced total number of and ADR for conventional adenomas in the left colorectum than did those in the placebo group. As well as a reduction in total colorectal adenoma number, participants randomly assigned to receive aspirin had a reduced number of adenomas in the right colon, particularly for serrated adenomas, but also a reduced risk of conventional colorectal adenomas.

The ADR was chosen as the primary outcome because previous polyp prevention trials have used this binary measure of colorectal adenoma occurrence as the primary endpoint.^12^ The high ADR (61%) in the placebo group in patients who were high risk so-called polyp formers was anticipated,^21^ but is higher than that observed in previous polyp prevention trials that included a majority of patients at intermediate risk. Notably, previous aspirin RCTs with the highest placebo ADR (47·1% and 53·4%) have reported no significant risk reduction associated with aspirin,^12^ despite the overwhelming evidence for a colorectal cancer chemoprevention effect of aspirin.^13^ The high ADR in the seAFOod trial will also have been driven by uniformly excellent colonoscopy quality in the BCSP, in which the ADR is a key endoscopist performance indicator.^2^, ^22^ Therefore, use of ADR as a chemoprevention biomarker in high-risk cohorts with a high ADR, undergoing quality-assured colonoscopic assessment, should be questioned.

By contrast, previous RCTs in patients with familial adenomatous polyposis have reported efficacy on the basis of colorectal adenoma number.^10^ Colorectal adenoma number is recognised to predict future colorectal cancer incidence and mortality in observational studies.^23^ Reduction in colorectal adenoma multiplicity, similar to that observed in the seAFOod trial, has been reported in all of the three aspirin RCTs that used adenoma number as a secondary outcome.^16^, ^24^, ^25^ The observational evidence that aspirin reduces long-term colorectal cancer risk,^13^ combined with a reduction in colorectal adenoma number observed in previous polyp prevention trials that is similar to that seen in the seAFOod trial,^12^ suggests that the effect sizes for EPA and aspirin reported in this Article will translate into a clinically meaningful decrease in long-term colorectal cancer risk.

More recently, adenoma number has gained credence as a primary outcome measure in polyp prevention RCTs,^15^ driven by the increasing quality of colonoscopy and use of the mean number of adenomas per participant as an outcome in endoscopy quality-assurance studies.^22^ Therefore, we suggest that future chemoprevention RCTs utilise number of adenomas per participant as the primary outcome.

Chemopreventive activity of EPA against conventional colorectal adenomas (but not serrated lesions), is consistent with the known efficacy of the same dose of EPA–FFA in patients with familial adenomatous polyposis and rectal adenomas,^10^ which are conventional, dysplastic lesions. The seAFOod trial data concur with evidence from long-term follow-up of vascular RCTs that show that reduction in colorectal cancer incidence and mortality with aspirin is explained by an effect on proximal neoplasia, with less preventive efficacy against distal colorectal cancer.^13^ Two observational studies already support a beneficial effect of aspirin on serrated polyp risk.^26^, ^27^ The seAFOod trial data should prompt a paradigm shift towards stratified chemoprevention trials with analysis of colorectal adenoma subtype and tumour location outcomes. Differential preventive activity against conventional and serrated pathway lesions by EPA and aspirin has highlighted the relatively poor characterisation of the molecular phenotype of colorectal adenomas compared with colorectal cancer,^7^, ^9^ and the poor understanding of the mechanism of action of both agents. Subsequent mechanistic studies of EPA and aspirin should investigate differential activity in models of chromosomal instability, microsatellite instability, and CIMP carcinogenesis pathways.

Total and left-sided colorectal adenoma multiplicity was lower in the group receiving combined treatment with EPA and aspirin, consistent with efficacy of both agents on left-sided, conventional colorectal adenomas. A factorial trial design that assumes independent intervention effects can only detect a large interaction between EPA and aspirin. A key objective of future work will be to apply precision medicine principles to establish which individuals might gain most from chemoprevention with one or both agents, based on baseline colorectal adenoma characteristics alone or together with other mucosal biomarkers.^9^

The seAFOod trial became a unique opportunity to compare the tissue bioavailability and tolerability of two EPA formulations of the same FFA dose in an RCT. There appeared to be no meaningful difference in compliance, tissue EPA incorporation, or tolerability between the FFA and triglyceride formulations, contrasting with many previous short-term studies of different omega-3 PUFA formulations.^28^ We confirmed the excellent safety profile of EPA and aspirin with an excess of mild-to-moderate gastrointestinal adverse events in EPA users, which is a consistent finding in omega-3 PUFA trials.^10^, ^28^ The excess of gastrointestinal symptoms in the group treated with EPA alone compared with the group treated with EPA and aspirin was unexpected, and requires validation and mechanistic explanation in parallel with further assessment of combination chemoprevention with both EPA and aspirin.

Omega-3 PUFA concentrations in red blood cells were similar to data from studies in other countries with relatively low dietary fish intake,^29^ with no evidence that trial participation altered dietary omega-3 PUFA intake. Despite self-reported compliance being excellent by capsule counting, the increase in EPA concentrations in red blood cells observed in active EPA users was highly variable, a phenomenon which has been seen in previous omega-3 PUFA studies.^30^ Detailed analysis of the relationship between individual baseline omega-3 PUFA concentrations, dietary omega-3 PUFA intake, and response to EPA dosing versus colorectal adenoma number is now required.

No evidence of substantial conversion of EPA to docosahexaenoic acid either in red blood cell membranes or rectal mucosa was seen. Therefore, the trial data do not support EPA as a universal omega-3 PUFA donor in the colorectum and no conclusions can be drawn from the trial about the efficacy of mixed EPA and docosahexaenoic acid formulations, which represent the majority of prescription and nutritional supplement omega-3 PUFA formulations.

Strengths of the trial include excellent, quality-assured colonoscopy performance and histological reporting, and uniform, protocol-driven care in the BCSP.^2^, ^22^ We chose to study a high-risk surveillance cohort to reduce study duration and costs. We have previously justified the use of a 1 year endpoint on the basis of positive findings at 1 year colonoscopy that mirror longer follow-up in previous aspirin polyp prevention trials,^12^ and evidence that colorectal adenoma outcomes after 1 year represent de-novo adenoma growth rather than solely missed lesions.^17^ The low number of advanced colorectal adenomas detected compared with previous RCTs is a limitation and probably relates to the short surveillance interval and the completeness of clearance of the screening colonoscopy in the BCSP. The male predominance reflects actual screening practice in the UK, as does the high prevalence of excess bodyweight in those with high-risk neoplasia.^2^ The trial only reached 83% of its recruitment target due primarily to a high screening failure, which included a higher-than-expected number of individuals who were unwilling to provide consent after BCSP screening. This finding has important implications for acceptability of long-term chemoprevention, even in high-risk cohorts, which requires further behavioural research. However, the reduced sample size still yielded sufficient precision to exclude a prespecified difference of 10% from placebo in the ADR for both EPA and aspirin.

In conclusion, the seAFOod Polyp Prevention trial has shown that the omega-3 PUFA EPA (2 g FFA per day) and aspirin (300 mg per day) did not reduce colorectal adenoma risk (as measured by the proportion of participants with at least one adenoma) at 1 year surveillance colonoscopy in individuals at high risk with colorectal neoplasia in the English BCSP. However, both agents had some chemopreventive efficacy on colorectal adenoma burden, as measured by a reduction in the mean number of adenomas per participant. The colorectal adenoma subtype-dependent and location-dependent specificity of EPA and aspirin are consistent with previous observations. Existing data on colorectal cancer risk reduction by aspirin suggest that the decrease in colorectal adenoma recurrence that we report for both agents is likely to translate into a clinically meaningful decrease in long-term colorectal cancer risk.
