# Supplementary material for: Eicosapentaenoic acid and aspirin, alone and in combination, for the prevention of colorectal adenomas (seAFOod Polyp Prevention trial): a multicentre, randomised, double-blind, placebo-controlled, 2 × 2 factorial trial
Source: Lancet. 2018 Dec 15;392(10164):2583–94. doi: 10.1016/S0140-6736(18)31775-6 (PMC6294731; doi:10.1016/S0140-6736(18)31775-6)
Supplement: Supplementary appendix [file mmc1.pdf]

THE LANCET

Supplementary appendix

This appendix formed part of the original submission and has been peer reviewed. We post it as supplied by the authors.

Supplement to: Hull MA, Sprange K, Hepburn T, et al. Eicosapentaenoic acid and aspirin, alone and in combination, for the prevention of colorectal adenomas (seAFOod Polyp Prevention trial): a multicentre, randomised, double-blind, placebo-controlled, 2 × 2 factorial trial. *Lancet* 2018; published online Nov 19. http://dx.doi.org/10.1016/S0140-6736(18)31775-6.

Web Appendix

Eicosapentaenoic acid and aspirin, alone and in combination, for the prevention of colorectal adenomas (seAFOod Polyp Prevention trial): a multicentre, randomised, double-blind, placebo-controlled, 2 x 2 factorial trial

Mark A Hull, Kirsty Sprange, Trish Hepburn, Wei Tan, Aisha Shafayat, Colin J Rees, Gayle Clifford, Richard F Logan, Paul M Loadman, Elizabeth A Williams, Diane Whitham, Alan A Montgomery, on behalf of the seAFOod Collaborative Group

The EPA-triglyceride formulation and stability testing

90% EPA-TG capsules obtained from Igennus Healthcare Nutrition (Cambridge, UK) each contained 574 mg of PUFAs, of which approximately 516 mg was re-esterified EPA triglycerides. This is equivalent to 400 mg EPA free fatty acids, thereby providing EPA-FFA dose equivalence to the EPA-FFA formulation by daily intake of five capsules. 90% EPA-TG capsules also contained 3.9% (of the total area under chromatographic peaks measured by gas-chromatography-mass spectrometry [GC-MS]) arachidonic acid, as well as small amounts (<1%) of other PUFAs. 90% EPA-TG contained 0.5% C16:4ω3, but this ω-3 PUFA was not detectable in the EPA-FFA formulation (in-house data).

The Medicines and Healthcare Products Regulatory Authority (MHRA) Clinical Trials Authorisation for 90% EPA-TG that was obtained during the trial stipulated a programme of stability testing in order to meet manufacturing Quality Assurance requirements for a CTIMP. A programme of 3 monthly accelerated (30°C 65% relative humidity [RH]) and standard (25°C 60% RH) stability testing (ALS Food & Pharmaceutical) of the peroxide value (POV), anisidine value (pAV) and derived TOTOX value, as well as full PUFA analysis by GC-MS, was performed. Rolling stability testing provided a continuous extension of shelf life for 90% EPA-TG until June 2016 when capsule IMP use finished, as per the maximum approved shelf-life (three years) approved by the MHRA.

Measurement of dietary fish intake

The validated EPIC short Food Frequency Questionnaire (FFQ) was used, which is a semi-quantitative, paper-based FFQ that includes 130 food items and supplementary questions about use of fat, milk, cooking methods, salt and supplement use over the preceding 12 months (McKeown NM, Day NE, Welch AA, et al. Use of biological markers to validate self-reported dietary intake in a random sample of the European Prospective Investigation into Cancer United Kingdom Norfolk cohort. *Am J Clin Nutr* 2001;**74**:188-96). The primary purpose of the FFQ was to determine if there was any change in dietary marine ω-3 PUFA intake during trial participation. Since fish is the primary source of bioactive ω-3 PUFAs EPA and DHA, the reported consumption of fish was analysed before and after intervention with IMP.

Total fish and oily fish consumption were considered separately. Six FFQ food item variables were used for the analysis of total fish (fried fish, fish fingers, white fish, oily fish, shell fish, roe) Consumption of oily fish was based on a single food variable (oily fish). Participants indicated the frequency of food consumption ranging from 'Never or <1/month' to '6 times/day'). This was recoded into frequency per day (see table A). The consumption of total fish/day was calculated by summing the reported fish consumption for each of the 6 fish variables.

Table A

FFQ category	Frequency per day
Never or less than once / month	0
1 – 3 per month	0.07
Once a week	0.14
2-4 per week	0.43
5-6 per week	0.79
Once per day	1
2-3 per day	2.5
4-5 per day	4.5
6+ per day	6

The data were used to code participants into 4 categories (never, low, middle or high) based on weekly consumption of total fish and oily fish (table B). Categorisation of weekly fish

consumption was based on the Scientific Advisory Committee on Nutrition (SACN) recommendation to consume 2 portions of fish per week, one of which should be oily (Scientific Advisory Committee on Nutrition/Committee on Toxicity. *Advice on Fish Consumption: Benefits & Risks*. 2004).

Table B

**Total fish**		
	**Category**	**Reported frequency of consumption**
	Never	0
	Low	Less than 1 portion per week (0.01-0.13/day)
	Middle	1-2.99 portions per week (0.14-0.429/day)
	High	3 or more portions per week (0.43-HIGHEST/day)
**Oily fish**		
	**Category**	**Reported frequency of consumption**
	Never	0
	Low	Less than 1 portion per week (0.07/day)
	Middle	1 portion per week (0.14/day)
	High	3 or more portions per week (0.43-HIGHEST/day)

Additional outcome data

Table S1: Compliance with trial medication

	placebo (n=176)	EPA (n=178)	aspirin (n=176)	EPA+aspirin (n=177)
**Participants who never started IMP**	**4 (2)[1]**	**2 (1)**	**4 (2)**	**7 (4)**
Never started either	1 (1)	1 (1)	2 (1)	6 (3)
Never started capsules but started tablets	1 (1)	0	1 (1)	1 (1)
Never started tablets but started capsules	2 (1)	1 (1)	1 (1)	0
**Percentage capsules taken[2]**				
Mean[SD]	95 [14]	94 [13]	95 [12]	96 [15]
Median[IQR]	100 [97, 100]	99 [96, 100]	100 [97, 100]	100 [97, 100]
Min, max	20, 100	27, 101	40, 123	10, 197
**Percentage tablets taken[2]**				
Mean[SD]	97 [9]	97 [9]	97 [6]	97 [8]
Median[IQR]	100 [98, 100]	99 [97, 100]	100 [98, 100]	100 [98, 100]
Min, max	71, 196	27, 101	55, 100	10, 100

[1]Figures in brackets are % values

[2]Percentage compliance with medication was calculated based on the total number of calculated doses when participants were taking the medication, as a proportion of the total number of expected doses. The period in which participants should have taken medication was between randomisation and the first surveillance colonoscopy. If participants discontinued and did not have the surveillance colonoscopy then the period was between randomisation and the last date known to be taking medication.

Table S2: Concomitant medication

	placebo (n=176)	EPA (n=178)	aspirin (n=176)	EPA+aspirin (n=177)
**Participants starting prescribed medication during the trial**	55 (31)[1]	43 (24)	42 (24)	42 (24)
**Number of participants starting**[2]				
Statin	9	12	6	6
Calcium + Vitamin D	0	1	0	1
Metformin	3	2	3	1
Proton Pump Inhibitor	12	13	8	5
Aspirin	5	6	1	3
Fish oil	1	1	0	1
Non-aspirin NSAID	23	9	16	13
Other	23	17	17	25

[1]Figures in brackets are % values
[2]Not mutually exclusive, some participants reported more than one

Table S3: Dietary fish and other seafood intake at baseline and at the end of the intervention

	placebo (n=176)	EPA (n=178)	aspirin (n=176)	EPA+aspirin (n=177)
**Completion of baseline FFQ**	174 (99)[1]	176 (99)	173 (98)	177 (100)
**Total fish intake at baseline**				
Never	11 (6)	7 (4)	4 (2)	4 (2)
Low	10 (6)	13 (7)	9 (5)	12 (7)
Medium	97 (56)	92 (52)	109 (63)	97 (55)
High	44 (25)	52 (30)	44 (25)	51 (29)
Missing[2]	12 (7)	12 (7)	7 (4)	13 (7)
**Oily fish intake at baseline**				
Never	42 (24)	52 (30)	43 (25)	48 (27)
Low	69 (40)	52 (30)	53 (31)	54 (31)
Medium	42 (24)	46 (26)	52 (31)	41 (23)
High	20 (11)	23 (13)	22 (13)	32 (18)
Missing[2]	1(1)	3 (2)	3(2)	2 (1)
**Completion of FFQ at 12 mo**	146 (89)	135 (76)	143 (81)	138 (78)
**Total fish intake at 12 mo**				
Never	11 (8)	3 (2)	3 (2)	3 (2)
Low	12 (8)	7 (5)	8 (6)	6 (4)
Medium	82 (56)	74 (55)	85 (59)	77 (56)
High	32 (22)	41 (30)	34 (24)	45 (33)
Missing[2]	9 (6)	10 (7)	13 (9)	7 (5)
**Oily fish intake at 12 mo**				
Never	42 (29)	38 (28)	36 (25)	33 (24)
Low	46 (32)	37 (28)	40 (28)	40 (29)
Medium	38 (26)	39 (29)	47 (33)	35 (26)
High	17 (12)	16 (12)	15 (10)	26 (19)
Missing[2]	3(2)	5 (4)	5 (3)	4 (3)

[1] Figures in brackets are % values
[2] FFQ was received, however data were not evaluable

Table S4: RBC and rectal mucosal EPA levels in participants who received active capsule IMP as EPA-FFA or EPA-TG

	EPA-FFA (n=212)[1]	EPA-TG (n=143)	Total (n=355)
**RBC EPA level**[2]			
**At baseline**			
Mean[SD]	0.6 [0.6]	0.5 [0.4]	0.6 [0.5]
Median[IQR]	0.5 [0.3, 0.8]	0.4 [0.3, 0.7]	0.5 [0.3, 0.8]
Min, max	0, 4.7	0, 1.8	0, 4.7
n	181	128	309
**At 6 months**			
Mean[SD]	2 [1.3]	1.9 [1.4]	1.9 [1.3]
Median[IQR]	1.9 [0.9, 2.9]	1.6 [0.9, 2.7]	1.8 [0.9, 2.8]
Min, max	0, 6.6	0, 8.2	0, 8.2
n	164	108	272
**At 12 months**			
Mean[SD]	1.9 [1.4]	1.6 [1.0]	1.8 [1.3]
Median[IQR]	1.7 [0.9, 2.8]	1.4 [0.8, 2.2]	1.6 [0.9, 2.4]
Min, max	0.1, 5.6	0, 4.9	0, 5.6
n	157	100	257
**RBC EPA absolute change from baseline at 6 months**			
Mean[SD]	1.3 [1.4]	1.3 [1.4]	1.3 [1.4]
Median[IQR]	1.3 [0.3, 2.3]	1.1 [0.4, 2.2]	1.2 [0.3, 2.3]
Min, max	-4.5, 6.1	-1.7, 7.2	-4.5, 7.2
n	151	103	254
**RBC EPA absolute change from baseline at 12 months**			
Mean[SD]	1.2 [1.4]	1.1 [1.1]	1.2 [1.2]
Median[IQR]	1.0 [0.1, 2.0]	0.9 [0.3, 1.6]	1.0 [0.2, 1.9]
Min, max	-3.0, 5.2	-0.8, 4.2	-3.0, 5.2
n	143	95	238
**Rectal mucosal EPA level at 12 months**			
Mean[SD]	1.3 [1.0]	1.8 [1.1]	1.5 [1.1]
Median[IQR]	1.1 [0.6, 1.7]	1.6 [1.0, 2.3]	1.3 [0.7, 2.0]
Min, max	0, 5.2	0.4, 5.0	0, 5.2
n	153	96	249

[1]number of individuals randomised to each active EPA formulation
[2]PUFA data are % total fatty acids

Table S5: Red blood cell DHA, AA and EPA/AA ratio levels in individuals who received capsule IMP as EPA-FFA or EPA-TG (active EPA groups only)

	EPA-FFA (N=212)	EPA-TG (N=143)	Total (N=355)
**DHA**[1]			
**baseline**			
Mean[SD]	2.1 [1.4]	2.3 [1.8]	2.2 [1.5]
Median[IQR]	2 [1, 2.9]	2.1 [1, 3]	2.0 [1, 2.9]
Min, max	0.1, 7.7	0.1, 9.7	0.1, 9.7
N	181	128	309
**6 months**			
Mean[SD]	2.0 [1.2]	1.6 [1.2]	1.8 [1.2]
Median[IQR]	2.0 [1.0, 2.5]	1.5 [0.6, 2.0]	1.7 [0.8, 2.4]
Min, max	0, 7.5	0.1, 6.8	0, 7.5
N	164	108	272
**12 months**			
Mean[SD]	2.2 [1.5]	1.9 [1.1]	2.1 [1.4]
Median[IQR]	2.2 [1.3, 2.8]	1.9 [1.2, 2.3]	1.9 [1.2, 2.7]
Min, max	0.1, 14.4	0, 6.6	0, 14.4
N	157	100	257
**Change at 6 months**			
Mean[SD]	-0.2 [1.4]	-0.5 [1.6]	-0.3 [1.5]
Median[IQR]	-0.2 [-0.9, 0.4]	-0.4 [-1.2, 0.3]	-0.2 [-1.0, 0.3]
Min, max	-4.5, 5.4	-8.6, 3.7	-8.6, 5.4
N	151	103	254
**Change at 12 months**			
Mean[SD]	-0.1 [1.4]	-0.3 [1.7]	-0.2 [1.5]
Median[IQR]	-0.2 [-0.9, 0.8]	-0.3 [-1.4, 0.7]	-0.2 [-1.1, 0.7]
Min, max	-4.2, 4.3	-7.7, 2.8	-7.7, 4.3
N	143	95	238
**AA**			
**baseline**			
Mean[SD]	6.3 [3.9]	7.0 [4.9]	6.6 [4.4]
Median[IQR]	5.8 [3.7, 8.2]	6.3 [3.9, 9.0]	5.9 [3.7, 8.3]
Min, max	0.4, 27.9	0.5, 32.1	0.4, 32.1
N	181	128	309
**6 months**			
Mean[SD]	5.6 [2.9]	5.5 [3.3]	5.5 [3.1]
Median[IQR]	5.8 [3.5, 7.2]	4.7 [2.7, 7.8]	5.4 [2.9, 7.2]
Min, max	0.1, 18.5	0.3, 16.6	0.1, 18.5
N	164	108	272
**12 months**			
Mean[SD]	6.1 [3.7]	6.1 [3.1]	6.1 [3.4]
Median[IQR]	6 [3.8, 7.7]	5.8 [3.8, 7.9]	5.9 [3.8, 7.7]
Min, max	0.3, 26.1	0, 13.3	0, 26.1
N	157	100	257
**Change at 6 months**			
Mean[SD]	-0.8 [3.8]	-1.1 [4.8]	-0.9 [4.2]
Median[IQR]	-0.9 [-2.6, 1.0]	-0.9 [-3.2, 1.9]	-0.9 [-3.0, 1.3]
Min, max	-14.6, 10.4	-25.9, 7.3	-25.9, 10.4
N	151	103	254

	EPA-FFA (N=212)	EPA-TG (N=143)	Total (N=355)
**Change at 12 months**			
Mean[SD]	-0.3 [4.1]	-0.8 [5.1]	-0.5 [4.5]
Median[IQR]	-.6 [-2.8, 2.1]	-0.6 [-3.9, 2.3]	-0.6 [-3.0, 2.2]
Min, max	-11.9, 13.1	-22.9, 7.9	-22.9, 13.1
N	143	95	238
**EPA/AA ratio**			
**At baseline**			
Mean[SD]	0.1 [0.1]	0.1 [0]	0.1 [0.1]
Median[IQR]	0.1 [0.1, 0.1]	0.1 [0.1, 0.1]	0.1 [0.1, 0.1]
Min, max	0, 0.6	0, 0.3	0, 0.6
N	181	128	309
**At 6 months**			
Mean[SD]	0.4 [0.2]	0.3 [0.2]	0.4 [0.2]
Median[IQR]	0.4 [0.2, 0.5]	0.3 [0.2, 0.4]	0.4 [0.2, 0.5]
Min, max	0, 0.8	0, 0.7	0, 0.8
N	164	108	272
**At 12 months**			
Mean[SD]	0.3 [0.2]	0.3 [0.1]	0.3 [0.2]
Median[IQR]	0.3 [0.2, 0.4]	0.2 [0.2, 0.4]	0.3 [0.2, 0.4]
Min, max	0, 0.8	0, 0.7	0, 0.8
N	157	100	257
**Change at 6 months**			
Mean[SD]	0.3 [0.2]	0.3 [0.2]	0.3 [0.2]
Median[IQR]	0.3 [0.1, 0.4]	0.3 [0.1, 0.4]	0.3 [0.1, 0.4]
Min, max	-0.5, 0.7	-0.1, 0.6	-0.5, 0.7
N	151	103	254
**Change at 12 months**			
Mean[SD]	0.2 [0.2]	0.2 [0.1]	0.2 [0.2]
Median[IQR]	0.2 [0.1, 0.3]	0.2 [0.1, 0.3]	0.2 [0.1, 0.3]
Min, max	-0.3, 0.7	-0.1, 0.6	-0.3, 0.7
N	143	95	238

[1]PUFA data are % total fatty acids

Table S6: Rectal mucosal PUFA levels at 12 months in individuals who received capsule IMP as EPA-FFA or EPA-TG (active EPA groups only)

	EPA-FFA (N=212)	EPA-TG (N=143)	Total (N=355)
**EPA**[1]			
Mean[SD]	1.3 [1.0]	1.8 [1.1]	1.5 [1.1]
Median[IQR]	1.1 [0.6, 1.7]	1.6 [1.0, 2.3]	1.3 [0.7, 2.0]
Min, max	0, 5.2	0.4, 5	0, 5.2
N	153	96	249
**DHA**			
Mean[SD]	0.9 [0.6]	1.2 [0.4]	1.0 [0.5]
Median[IQR]	0.7 [0.5, 1.4]	1.2 [1.0, 1.4]	1.0 [0.6, 1.4]
Min, max	0.1, 4.3	0.4, 2.1	0.1, 4.3
N	153	96	249
**AA**			
Mean[SD]	4.4 [2.8]	5.8 [1.6]	5.0 [2.5]
Median[IQR]	3.5 [2.3, 6.3]	5.5 [4.7, 6.5]	5.0 [2.8, 6.4]
Min, max	0.3, 17.7	1.9, 11.4	0.3, 17.7
N	153	96	249
**EPA/AA ratio**			
Mean[SD]	0.3 [0.2]	0.3 [0.2]	0.3 [0.2]
Median[IQR]	0.3 [0.2, 0.4]	0.3 [0.2, 0.4]	0.3 [0.2, 0.4]
Min, max	0, 0.9	0.1, 0.8	0, 0.9
N	153	96	249

[1]PUFA data are % total fatty acids

Table S7: Sensitivity analyses of the total ADR with adjustment for repeat colonoscopy

	EPA *versus* no EPA estimate (95% CI)	aspirin *versus* no aspirin estimate (95% CI)
**Using per protocol population (Risk diff)**	-0.6 (-8.7, 7.4)	-0.6 (-8.7, 7.4)
**Multi-level model treating recruiting centre and site as random effects (Odds ratio)**	0.96 (0.68, 1.34)	0.97 (0.70, 1.34)
**Multiple imputation of missing data (Risk diff)**	0.1 (-7.3, 7.6)	-0.7 (-8.3, 6.8)
**Adjustment of baseline variables with imbalance[2] (Risk diff)**	-0.7 (-8.5, 7.1)	-0.7 (-8.5, 7.1)
**Adjustment of oily fish intake during the trial (Risk diff)**	-2.5 (-11.1, 6.0)	-2.4 (-11.0, 6.1)
**CACE analysis taking account of treatment adherence**		
Binary adherence (Risk diff)	-1.0 (-9.9, 7.9)	-0.7 (-8.5, 7.1)
Continuous adherence (Risk diff)	-1.2 (-10.0, 7.5)	-0.8 (-8.8, 7.1)
**Adjustment for EPA formulation (Risk diff)**	-0.9 (-8.8, 6.9)	-0.4 (-8.3, 7.4)
**Adjustment for baseline RBC EPA (Risk diff)**	-4.2 (-12.6, 4.2)	2.3 (-6.0, 10.6)

[1]Risk difference as %

[2]Adjusted for sex, whether diagnosed with diabetes, and smoking status.

Table S8 Analyses of primary outcome without adjustment of repeat colonoscopy

	EPA *versus* no EPA estimate (95% CI)	aspirin *versus* no aspirin estimate (95% CI)
**Primary analysis (Risk diff)**	0.7 (-6.8, 8.3)	-1.2 (-8.7, 6.3)
**Using per protocol population (Risk diff)**	1.0 (-6.7, 8.8)	-1.0%(-8.7, 6.7)
**Multi-level model treating recruiting centre and site as random effects (Odds ratio)**	1.03 (0.75, 1.42)	0.95 (0.69, 1.31)
**Multiple imputation of missing data (Risk diff)**	0.1 (-7.3, 7.6)	-0.7 (-8.3, 6.8)
**Adjustment of baseline variables with imbalance[1] (Risk diff)**	1.1 (-6.4, 8.6)	-1.5 (-9.0, 5.9)
**Adjustment of oily fish intake during the trial (Risk diff)**	-1.3 (-9.5, 6.9)	-2.7 (-10.9, 5.5)
**CACE analysis taking account of treatment adherence**		
Binary adherence (Risk diff)	0.8 (-7.1, 8.7)	-1.2 (-8.9, 6.4)
Continuous adherence (Risk diff)	0.4 (-7.3, 8.1)	-1.3 (-8.6, 6.4)
**Adjustment for EPA formulation (Risk diff)**	0.8 (-6.7, 8.3)	-1.1 (-8.3, 7.4)
**Adjustment for baseline RBC EPA (Risk diff)**	-2.1 (-10.2, 5.9)	1.7 (-6.3, 9.8)

[1]Adjusted for sex, whether diagnosed with diabetes, and smoking status.

Table S9: AEs classified by MedDRA system organ class

	placebo (n=176)	EPA (n=177)	aspirin (n=174)	EPA+aspirin (n=170)
Cardiac disorders[1]	2	1	0	1
Congenital, familial and genetic disorders	0	1	0	0
Ear and labyrinth disorders	1	1	0	1
Eye disorders	0	0	0	1
Gastrointestinal disorders	85	146	86	68
General disorders and administration site conditions	6	2	2	4
Hepatobiliary disorders	0	2	0	0
Immune system disorders	1	1	1	0
Infections and infestations	12	14	15	14
Injury, poisoning and procedural complications	6	5	11	7
Investigations	1	3	0	3
Metabolism and nutrition disorders	0	1	3	0
Musculoskeletal and connective tissue disorders	13	9	10	12
Neoplasms benign, malignant and unspecified (incl. cysts and polyps)	3	0	3	1
Nervous system disorders	6	6	7	4
Psychiatric disorders	2	0	0	1
Renal and urinary disorders	2	1	5	4
Reproductive system and breast disorders	2	0	0	1
Respiratory, thoracic and mediastinal disorders	3	9	3	3
Skin and subcutaneous tissue disorders	9	4	5	3
Social circumstances	1	0	0	0
Surgical and medical procedures	2	2	0	3
Vascular disorders	3	3	0	1

[1] Event-level data

Table S10: Gastrointestinal AEs by MedDRA preferred term, treatment arm and EPA formulation

	placebo		EPA		aspirin		EPA+aspirin	
	**FFA**	**TG**	**FFA**	**TG**	**FFA**	**TG**	**FFA**	**TG**
**n in safety population**	**109**	**167**	**107**	**70**	**99**	**75**	**99**	**71**
**n (%) reporting ≥ one GI AE**	**38 (35)**	**13 (19)**	**43 (40)**	**24 (34)**	**24 (24)**	**20 (27)**	**25 (25)**	**22 (31)**
Abdominal discomfort	2	0	2	3	0	2	0	0
Abdominal distension	1	0	0	1	0	0	0	2
Abdominal pain	9	3	14	13	6	1	3	2
Abdominal pain lower	3	0	2	0	0	0	0	0
Abdominal pain upper	3	0	4	1	1	0	2	1
Anal haemorrhage	2	0	1	0	0	0	0	0
Anal inflammation	0	0	0	0	0	1	0	0
Anal pruritus	0	0	0	1	0	0	0	0
Breath odour	4	0	3	0	0	1	1	0
Change of bowel habit	0	0	0	0	1	1	0	0
Constipation	3	0	5	1	2	4	6	2
Defaecation urgency	0	1	3	0	0	0	0	0
Diarrhoea	6	6	23	15	11	9	8	2
Dyspepsia	14	4	8	7	6	8	5	11
Epigastric discomfort	0	0	0	0	0	0	0	1
Eructation	1	0	2	0	0	0	0	3
Faeces discoloured	2	0	1	1	0	0	0	0
Flatulence	3	3	2	2	1	1	1	0
Frequent bowel movements	3	0	1	0	0	0	1	0
Gastric haemorrhage	0	0	1	0	0	0	0	0
Gastritis	0	0	1	0	0	1	0	0
Gastrointestinal sounds abnormal	0	0	0	0	0	0	1	0
Gastro-oesophageal reflux disease	4	2	1	0	0	4	0	3

	placebo		EPA		aspirin		EPA+aspirin	
	**FFA**	**TG**	**FFA**	**TG**	**FFA**	**TG**	**FFA**	**TG**
**n in safety population**	**109**	**167**	**107**	**70**	**99**	**75**	**99**	**71**
**n (%) reporting ≥ one GI AE**	**38 (35)**	**13 (19)**	**43 (40)**	**24 (34)**	**24 (24)**	**20 (27)**	**25 (25)**	**22 (31)**
Gingival polyp	1	0	0	0	0	0	0	0
Haematochezia	0	0	1	0	4	1	0	0
Haemorrhoidal haemorrhage	0	0	1	0	0	2	0	0
Haemorrhoids	0	0	0	0	1	0	0	1
Hiatus hernia	0	0	1	0	1	1	0	0
Melaena	0	0	1	0	0	1	0	0
Mouth haemorrhage	0	0	0	0	1	0	0	0
Nausea	0	3	5	6	2	1	5	2
Oesophagitis	0	0	1	0	0	0	0	0
Proctalgia	0	0	1	0	0	0	0	0
Rectal haemorrhage	1	1	2	2	4	3	4	0
Rectal tenesmus	0	0	1	0	0	0	0	0
Retching	0	0	0	0	0	0	0	1
Tongue discolouration	0	0	1	0	0	0	0	0
Tongue eruption	0	0	1	0	0	0	0	0
Tongue haemorrhage	0	0	0	0	1	0	0	0
Vomiting	0	0	2	1	2	0	0	0

Table S11: Summary of GI Adverse Events[1]

	placebo		EPA		aspirin		EPA+aspirin	
	**FFA**	**TG**	**FFA**	**TG**	**FFA**	**TG**	**FFA**	**TG**
**n in safety population**	**109**	**167**	**107**	**70**	**99**	**75**	**99**	**71**
**n(%) reporting ≥ one GI AE**	**38(35)**	**13(19)**	**43(40)**	**24(34)**	**24(24)**	**20(27)**	**25(25)**	**22(31)**
Diarrhoea[2]	9	7	27	15	11	9	9	2
UGI symptoms	19	9	21	15	11	15	11	18
Lower abdominal symptoms	18	3	20	17	7	3	4	5
Eructation/halitosis	5	0	5	0	0	1	1	3
Other	11	4	19	7	15	14	12	3

[1]Summary categories defined by the Chief Investigator with derived from the MedDRA preferred terms in table S8
[2]Event-level data

Table S12: Adverse drug reactions classified by MedDRA system organ class

	placebo (n=176)	EPA (n=177)	aspirin (n=174)	EPA+aspirin (n=170)
**n (%) reporting ≥ one adverse drug reaction**	**38 (22)**	**57 (32)**	**41 (24)**	**43 (25)**
**Total number of adverse drug reactions**	**63**	**119**	**83**	**63**
Gastrointestinal disorders	58	110	69	46
frequency of GI adverse drug reactions per participant				
1	22	26	16	20
2	7	13	10	11
3	4	7	4	0
4	1	4	4	1
5	0	3	1	0
6	1	1	0	0
General disorders and administration site conditions	1	0	0	4
Infections and infestations	0	0	0	1
Injury, poisoning and procedural complications	0	1	3	4
Investigations	0	0	0	1
Metabolism and nutrition disorders	0	1	1	0
Musculoskeletal and connective tissue disorders	1	0	0	0
Nervous system disorders	0	3	4	3
Psychiatric disorders	0	0	0	1
Renal and urinary disorders	0	0	3	2
Respiratory, thoracic and mediastinal disorders	0	2	2	1
Skin and subcutaneous tissue disorders	3	2	1	0

Table S13: SAEs classified by MedDRA system organ class

	placebo (n=176)	EPA (n=177)	aspirin (n=174)	EPA+aspirin (n=170)
**n (%) reporting ≥ one SAE**	**13 (7)**	**12 (7)**	**12 (7)**	**5 (3)**
**Total number of SAEs**	**16**	**16**	**17**	**6**
Cardiac disorders	1	6	2	0
Gastrointestinal disorders	2	2	5	0
General disorders and administration site conditions	1	1	0	1
Hepatobiliary disorders	0	1	0	0
Infections and infestations	3	2	2	1
Injury, poisoning and procedural complications	0	0	1	0
Investigations	0	0	1	0
Neoplasms benign, malignant and unspecified (incl cysts and polyps)	4	2	0	2
Nervous system disorders	1	0	2	0
Psychiatric disorders	2	0	0	0
Respiratory, thoracic and mediastinal disorders	0	2	1	1
Vascular disorders	2	0	3	1

Table S14: Colorectal adenoma size

	placebo	EPA	aspirin	EPA+aspirin
Number of participants with mean colorectal adenoma size available at exit colonoscopy	100	97	100	97
Colorectal adenoma size (mm)[1]				
Mean[SD]	3.7 [2.4]	3.3 [2.2]	4.2 [3.3]	3.5 [2.3]
Median[IQR]	3.0 [2.0, 4.4]	3.0 [2.0, 4.0]	3.5 [2.3, 5.0]	3.0 [2.0, 3.5]
Min, max	1, 15	1, 15	1, 28.5	1, 12
	**EPA *versus* no EPA**		**aspirin *versus* no aspirin**	
Adjusted[2] difference in means	-0.47 (-1.04, 0.98)		0.42 (-0.14, 0.99)	

[1]The mean was calculated for each participant then the overall mean of the mean values was calculated for each arm.

[2]Adjusted by histology type (conventional/serrated) and recruiting site. Estimates are the mean difference taking into account multiple adenomas per participant.

TOTAL FISH

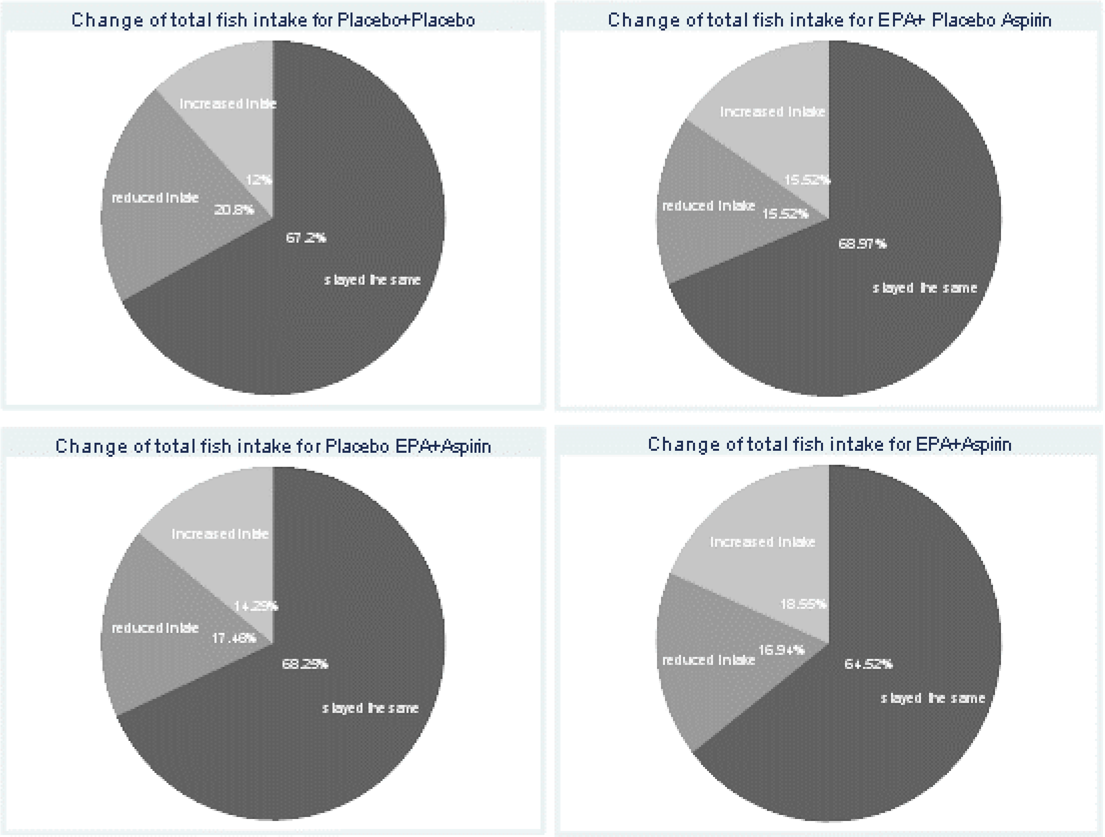

OILY FISH

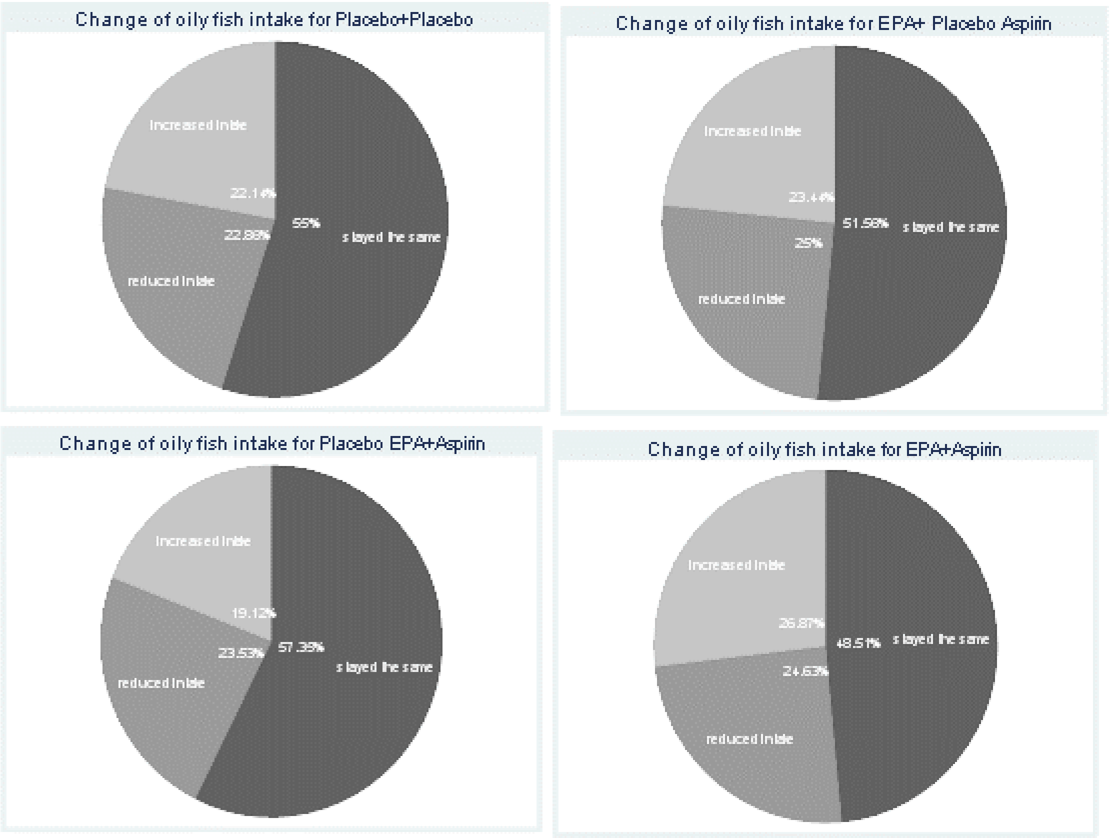

Figure S1: Change in total and oily fish intake category during the intervention period

A) Primary outcome (ADR)

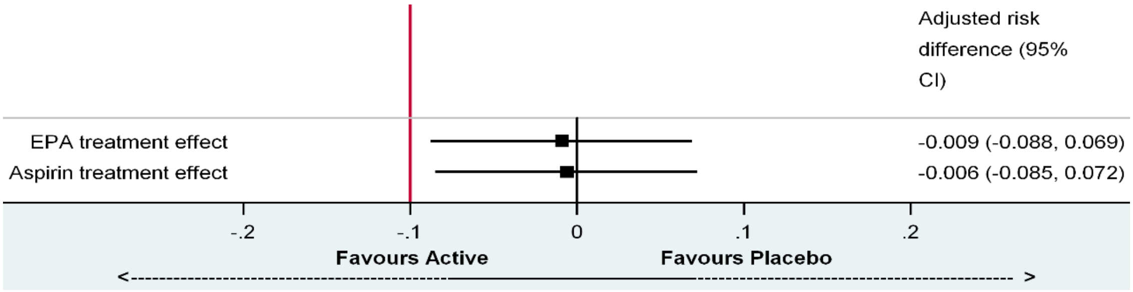

The red vertical line represents the pre-specified absolute target reduction in total ADR of 10%

B) Colorectal adenoma number outcomes

EPA

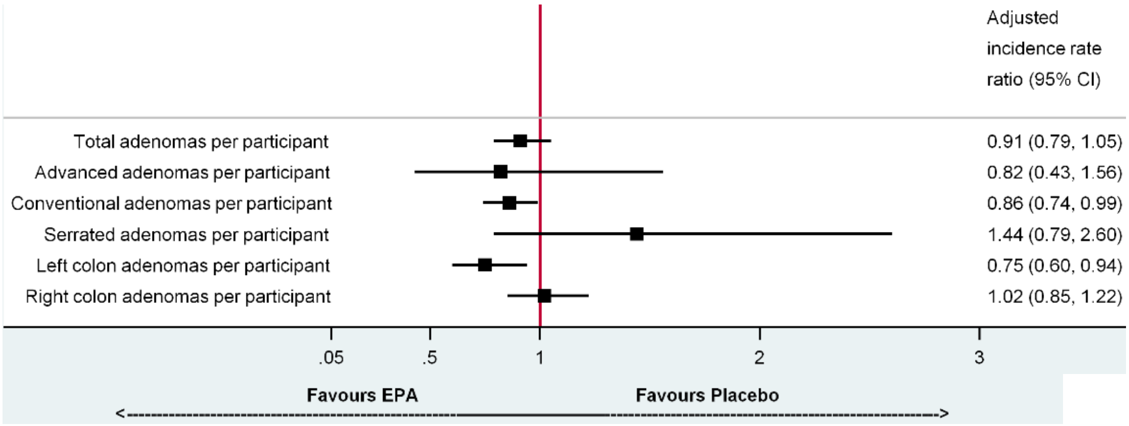

aspirin

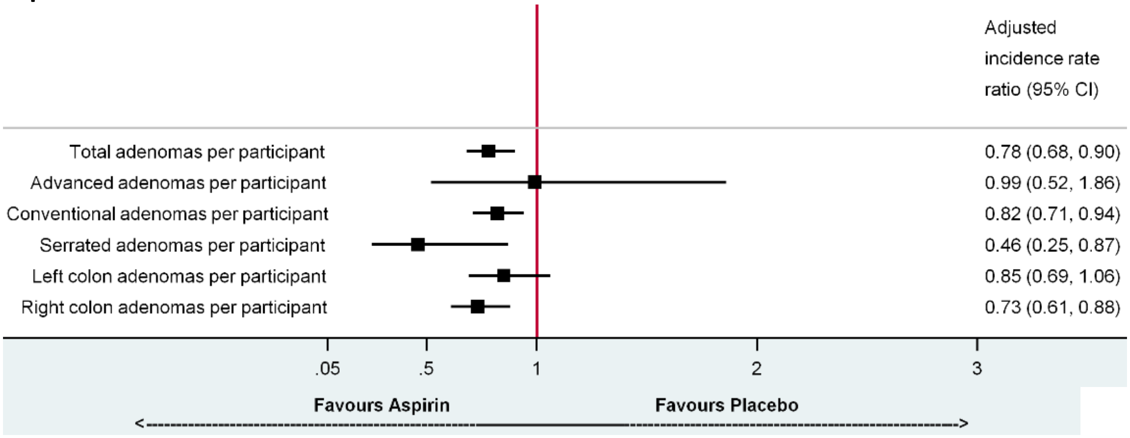

C) Secondary ADR outcomes

EPA

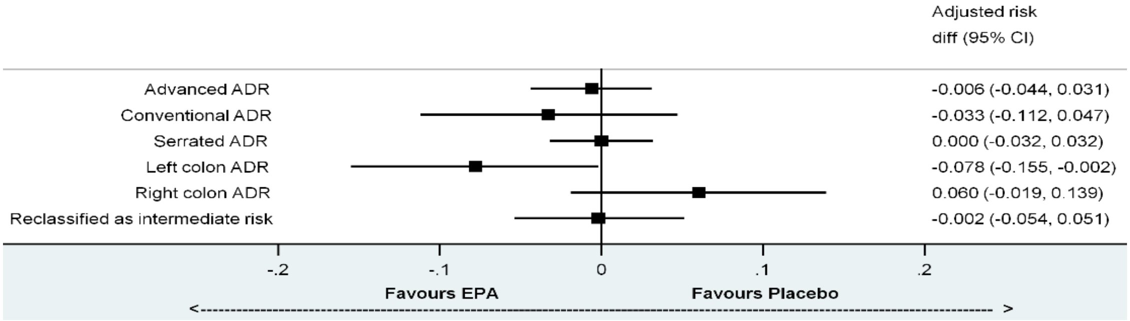

aspirin

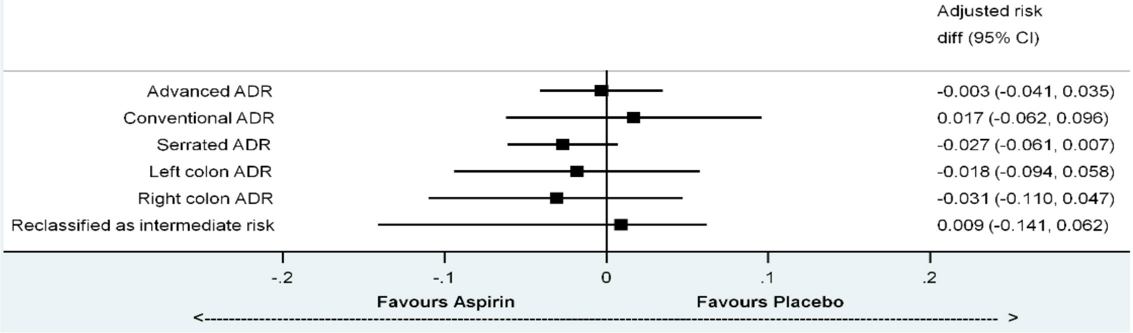

Figure S2: Forest plots for primary and secondary colorectal adenoma outcomes

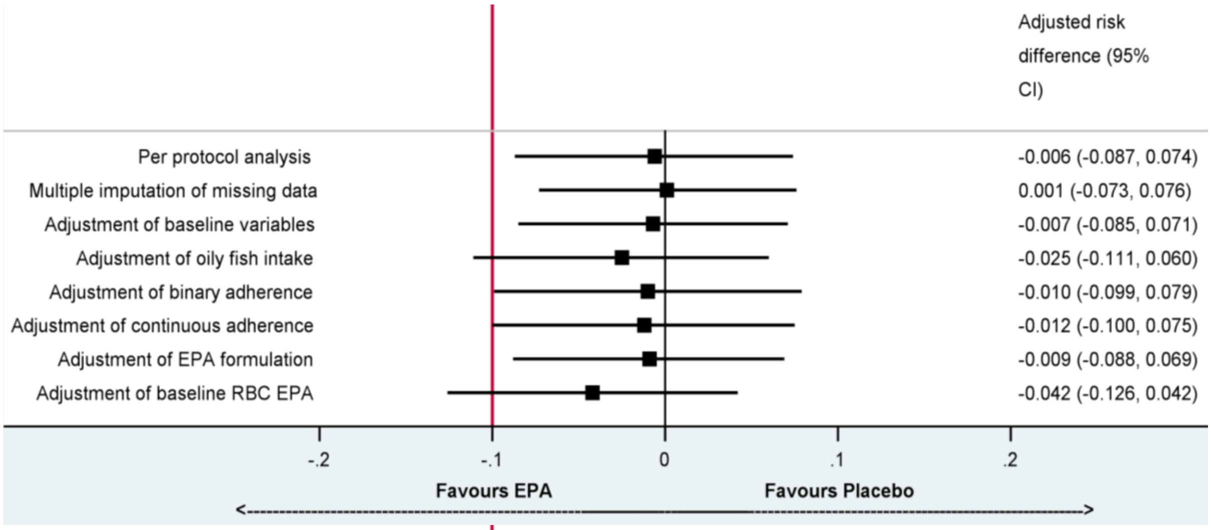

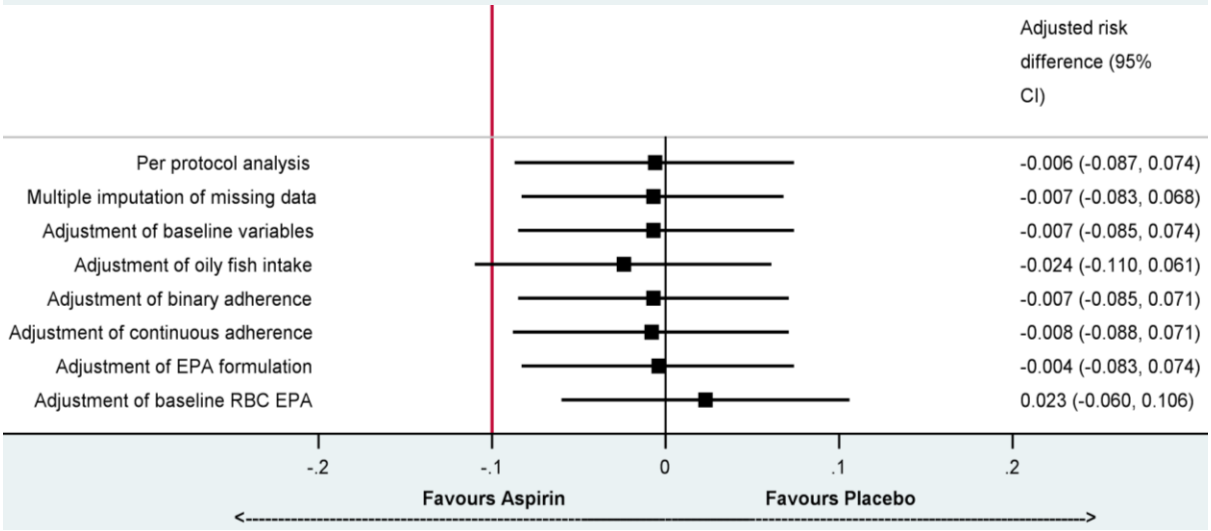

Multi-level model

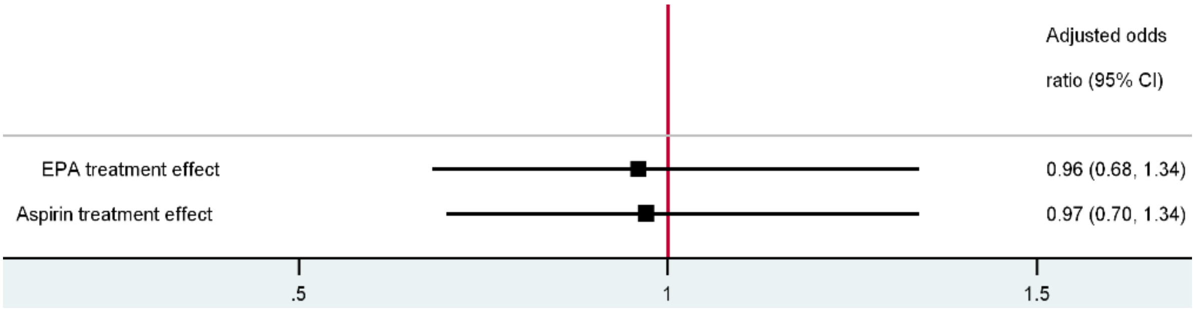

The multi-level model included both BCSP centre and site as random effects to account for organisational clustering of up to three BCSP sites in a single BCSP centre.
Results using the multi-level model are presented as the odds ratio, whereas all other sensitivity analyses are presented as the risk difference

Figure S3: Forest plots for the sensitivity analyses of the total ADR

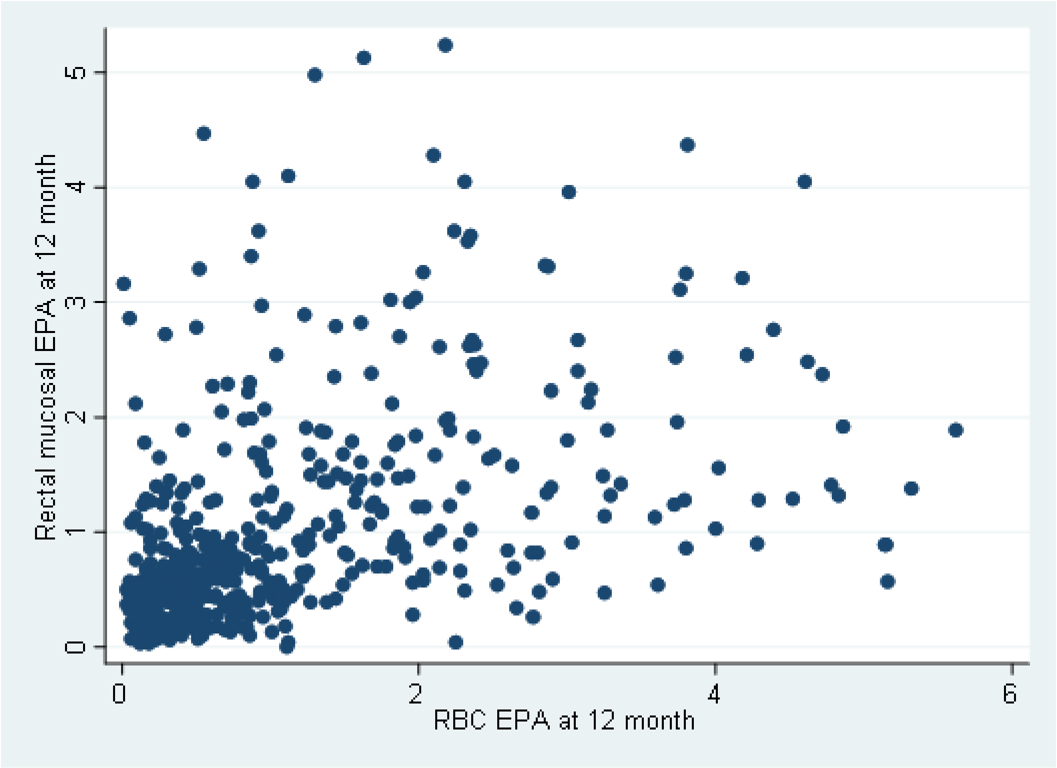

Figure S4: Scatter plot of the individual rectal mucosal EPA level against the corresponding RBC EPA level at 12 months

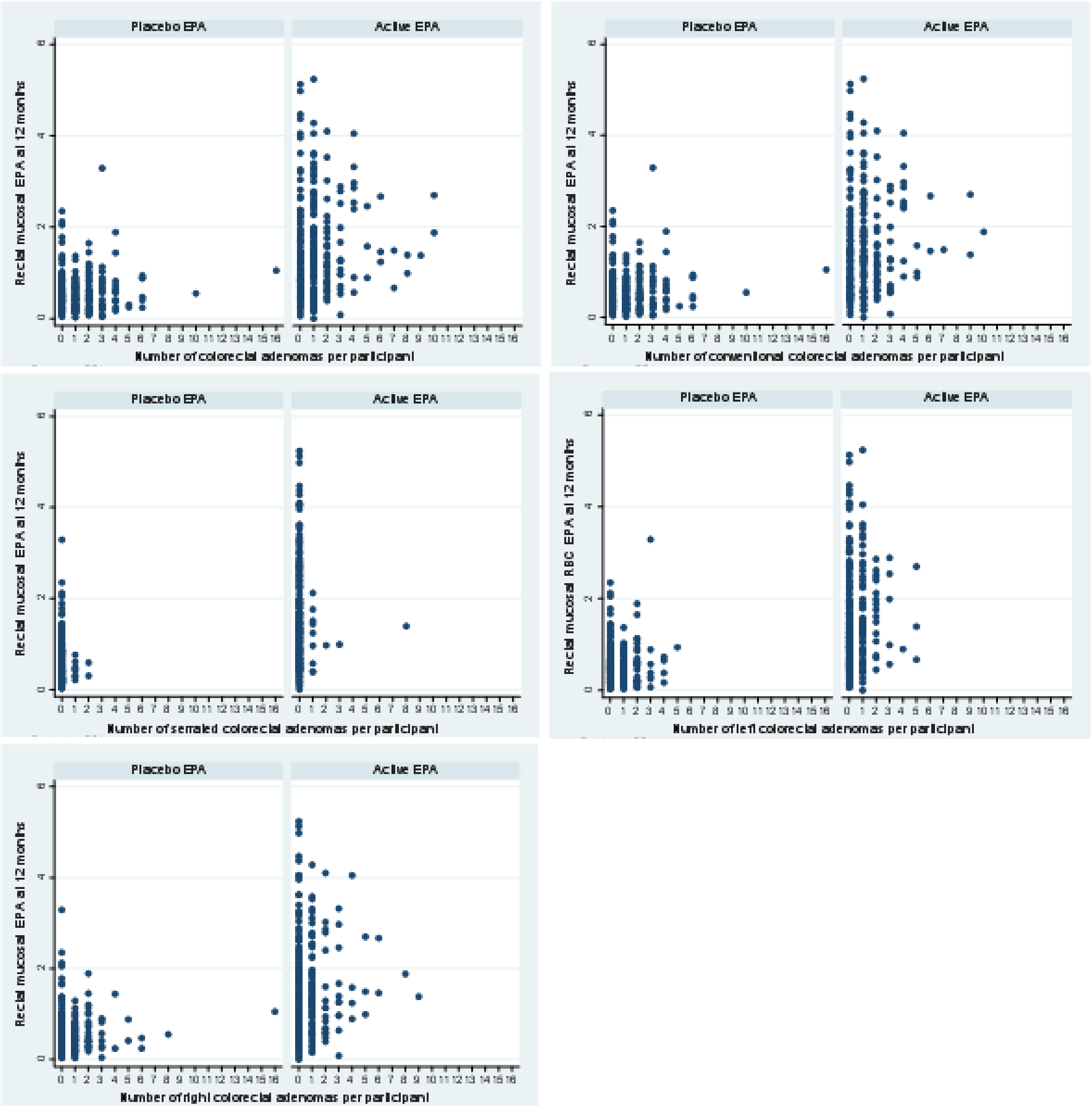

Figure S5: Rectal mucosal EPA measured at the end of the intervention against the number of colorectal adenomas per participant
